# Information sharing within a social network is key to behavioral flexibility—Lessons from mice tested under seminaturalistic conditions

**DOI:** 10.1126/sciadv.adm7255

**Published:** 2025-01-03

**Authors:** Maciej Winiarski, Anna Madecka, Anjaly Yadav, Joanna Borowska, Maria R. Wołyniak, Joanna Jędrzejewska-Szmek, Ludwika Kondrakiewicz, Lech Mankiewicz, Mayank Chaturvedi, Daniel K. Wójcik, Krzysztof Turzyński, Alicja Puścian, Ewelina Knapska

**Affiliations:** ^1^ Laboratory of Neurobiology of Emotions, Nencki-EMBL Partnership for Neural Plasticity and Brain Disorders–BRAINCITY, Nencki Institute of Experimental Biology of Polish Academy of Sciences, Warsaw, Poland.; ^2^ Laboratory of Neuroinformatics, Nencki Institute of Experimental Biology of Polish Academy of Sciences, Warsaw, Poland.; ^3^ Center for Theoretical Physics, Polish Academy of Sciences, Warsaw, Poland.; ^4^ Laboratory of Neurobiology, Nencki-EMBL Partnership for Neural Plasticity and Brain Disorders–BRAINCITY, Nencki Institute of Experimental Biology of Polish Academy of Sciences, Warsaw, Poland.; ^5^ The Kennedy Institute of Rheumatology, University of Oxford, Oxford, UK.; ^6^ Faculty of Management and Social Communication, Jagiellonian University, 30-348 Cracow, Poland.; ^7^ Institute of Theoretical Physics, Faculty of Physics, University of Warsaw, Warsaw, Poland.

## Abstract

Being part of a social structure offers chances for social learning vital for survival and reproduction. Nevertheless, studying the neural mechanisms of social learning under laboratory conditions remains challenging. To investigate the impact of socially transmitted information about rewards on individual behavior, we used Eco-HAB, an automated system monitoring the voluntary behavior of group-housed mice under seminaturalistic conditions. In these settings, male mice spontaneously form social networks, with individuals occupying diverse positions. We show that a rewarded group member’s scent affects the ability of conspecifics to search for rewards in familiar and novel environments. The scent’s impact depends on the animal’s social position. Furthermore, disruption of neuronal plasticity in the prelimbic cortex (PL) disrupts the social networks and animals’ interest in social information related to rewards; only the latter is blocked by the acute PL inhibition. This experimental design represents a cutting-edge approach to studying the brain mechanisms of social learning.

## Teaser

Social learning plays a key role in adjusting to the changing environment and shapes social networks.

## INTRODUCTION

Social structure resulting from kinship, friendship, and hierarchy among individuals is a hallmark of human society. Being a part of this structure is key for survival and reproduction (*1*, *2*). Similar structures are also observed in other social species, including rodents (*3*). Although living with conspecifics may generate substantial costs, such as competition for resources and parasite transmission, it also raises an individual’s evolutionary fitness in many ways, such as, for example, by providing valuable information about the environment. Social learning about environmental opportunities helps individuals to adjust to the rapidly changing circumstances without the need for firsthand experience, which can be costly as it requires foraging and thus energy expenditure (*4*–*7*). Yet, studying social learning and mechanisms of socially driven spreading of information within the social networks in group-housed mice is still not established under laboratory conditions.

Thus far, processing of information provided by others has almost exclusively been studied in simplified models using dyads or triads of interacting rodents (*8*–*10*). These studies showed that emotionally aroused animals transmit cues that can be detected and decoded by conspecifics (*11*). They also elucidated a set of neuronal circuits involved in the processing of social information, including networks within the prefrontal cortex (PFC) (*12*). In contrast, how learning scales up from individuals to social networks is yet to be determined. Moreover, we know very little about social learning under more complex conditions, i.e., in animals living in groups, using larger territory, and functioning within a social structure. Although difficult, such studies are of paramount importance because murine sociability is heavily dependent on population density, habitat size, and social structure (*13*).

Furthermore, most laboratory studies on social communication and learning rely on the assessment of direct interactions between individuals. However, under natural conditions, the life of mice, in particular their social life, is dominated by smell. Mice commonly use odors to detect and assess food and predators, recognize individuals, and evaluate sexual and social status (*14*–*17*). Notably, a major form of communication among mice is scent marking with urine that does not require direct contact between individuals (*18*, *19*).

Both rodent and human studies implicated the PFC in the processing of social information (*20*, *21*), discriminating the affective state of other individuals (*22*, *23*), maintaining social hierarchy (*24*), and social transmission of information about food safety (*25*). The PFC is known for the dynamic processes of neuronal plasticity. In some cognitive tasks dependent on the circuitry in both the PFC and subcortical areas, the refinement of the neuronal connectivity and activity is observed much faster in the former (*26*). These findings support the role of the PFC as a highly flexible and versatile structure for processing novel information in changing environments (*27*, *28*). Tissue inhibitors of metalloproteinases (TIMPs) are a family of endogenous proteins that affect synaptic plasticity, predominantly by inhibiting the enzymatic activity of matrix metalloproteinases (MMPs), in particular MMP-9 (*29*–*36*). TIMP-1 has been shown to disrupt neuronal plasticity in the PFC (*30*). Under physiological conditions, TIMP-1 plays a role in long-term potentiation (LTP) (*37*), a process crucial for forming memory on the cellular level. Here, we use nanoparticles (NPs) gradually releasing TIMP-1 to affect plasticity in the prelimbic part of the PFC (PL) and cause prolonged impairment in the updating of its neuronal connectivity, with a goal of disrupting social behavior. Furthermore, to differentiate between the effects of disrupting synaptic plasticity versus impairing neuronal activity, we counterbalance TIMP-1 experiments with the ultrapotent chemogenetic silencing studies and show diverse impact of the two.

Together, we illustrate the profound effects of social information about reward on animals’ behavior and their ability to adjust to changes in both familiar and novel environments. We show that mice—when provided with seminaturalistic conditions—spontaneously form social networks, effectively learn from social olfactory cues, and use such information to gain access to the reward. The animals display clear and stable individual differences in social interactions, and socially conveyed information has different effects on individual mice. Last, social learning and resulting adjustments of social structure are impaired when synaptic plasticity in the PL is disrupted, whereas only the former is deficient when the PL neurons are silenced.

## RESULTS

### The scent of a sucrose-rewarded mouse attracts other mice

To study how social information about the reward availability affects the behavior of individuals, we used Eco-HAB (*38*), an automated system for tracing social behavior and learning of mice living in a group under seminaturalistic conditions. Eco-HAB allows for measuring voluntary behaviors, with animals responding at their own pace, as well as for collecting data continuously and for much longer time than in classical tests.

**Fig. 1.**
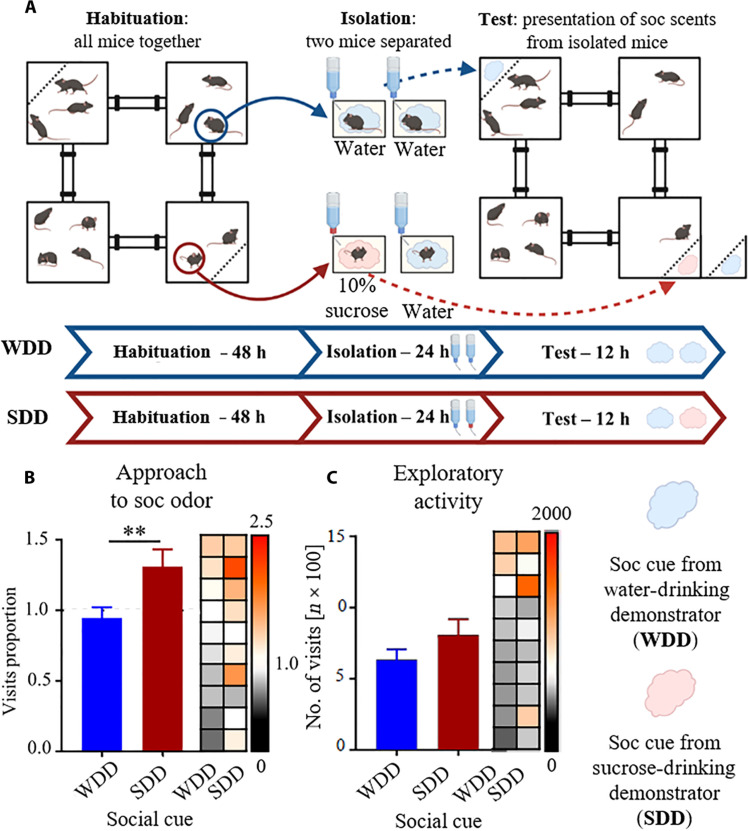
Social olfactory cues indicating a sucrose reward attract mice. (**A**) Schematic representation of the experimental design. Arrows indicate the relocation of the animals or their scents. h, hours. (**B**) Mice prefer the compartment where the bedding soaked with the scent of the SDD was presented over the compartment where the bedding soaked with the scent of the WDD was presented. Approach to social odor was calculated as a proportion of visits to the compartments containing olfactory cues [SDD-WDD (reward availability-neutral) to WDD-WDD (neutral-neutral)] divided by a respective parameter from the corresponding period 24 hours before the introduction of the social stimuli (see Materials and Methods). (**C**) Social olfactory cue indicating that reward availability does not change the exploratory activity. Total number of visits to all compartments of the Eco-HAB. Values for individual subjects are presented on the heatmap (right to the bar plots), squares in each row represent data for the same mouse, and columns represent trials, sorted by the control condition. Data in the bar graphs are shown as means ± SE. **P < 0.01. SDD, sucrose-drinking demonstrator; WDD, water-drinking demonstrator.

In the first round of experiments, after 48 to 72 hours of habituation to the Eco-HAB environment, 2 mice of 12 living together were separated from the group and put into individual cages for the next 24 hours. The separated mice provided a source of social olfactory information—bedding soaked with their urine and fecal matter—which was then presented to the rest of the group still inhabiting the Eco-HAB. The two scents were presented for 12 hours, in two distant, opposite spaces within the territory (Fig. 1A). To avoid mixing the scents, olfactory stimuli were placed behind the perforated separators so the mice could not touch the bedding. They had access only to the olfactory cues. Notably, the two separated mice did not come back to their original cohort in the testing phase, so there was no direct contact that might have resulted in the additional ways of communication between the conspecifics.

The same cohort of mice was subjected to two rounds of experimental procedures in the familiar environment. In the first, control round of testing, both separated mice got access to tap water while singly housed [water-drinking demonstrator (WDD)]. In the second round, the same two mice were separated, but this time, one of them got access to a highly rewarding 10% sucrose solution [sucrose-drinking demonstrator (SDD)], whereas the other drank water. We show that animals respond to the presentation of the social cue from a rewarded mouse in a familiar environment by adjusting their behavior. First, they display a strong interest in the bedding from a mouse having access to a 10% sucrose solution (a social cue from SDD) in comparison to the scent of a mouse having access to tap water (Fig. 1B). Notably, the overall exploratory activity of the mice is not changed (Fig. 1C), which suggests that the olfactory information affects social rather than general exploratory behavior. These results were confirmed by the replication experiment performed in another cohort of mice (fig. S1).

### Response to information about reward depends on the position in the social network

**Fig. 2.**
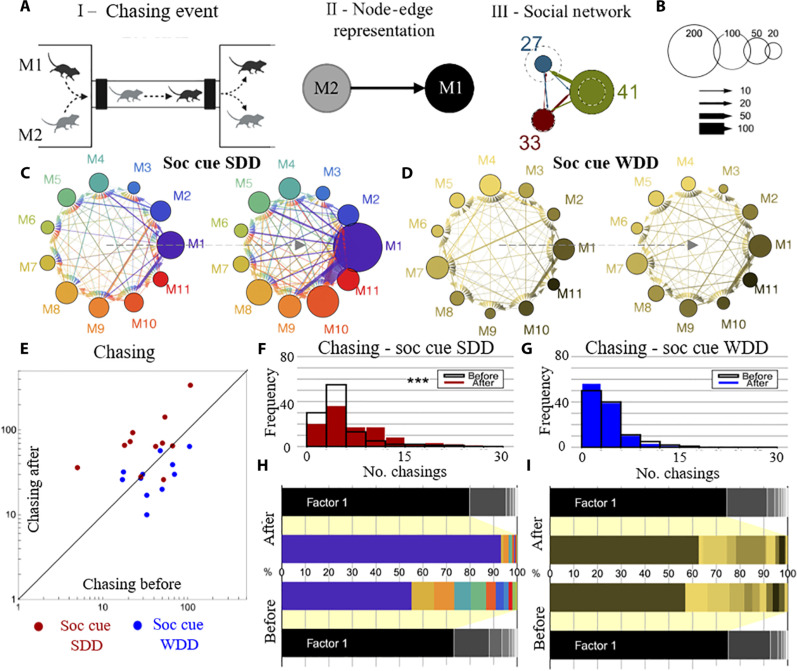
Mice form social networks based on individual differences in chasing behavior, and these social structures can change in response to social cues indicating the presence of a sucrose resource. (**A**) Social networks defined based on the patterns of chasing, (I) instances of mouse M2 trailing mouse M1. (II) Group’s social network is represented as a weighted, directed graph with nodes corresponding to individuals and edges to interactions between them. (III) Different colors represent the behavior of individual mice. (**B**) The radius of each node is proportional to the number of chasings performed by a corresponding mouse. The arrows are directed from a chaser to a chased individual; the thickness of an arrow is proportional to the number of chasings performed by a given mouse. (**C** to **G**) The presence of the social olfactory cues under the SDD condition increases chasing. Social network during the baseline and after the presentation of social cues indicating either the reward availability [(C) SDD] or the neutral stimuli [(D) WDD]. (E) Increase in chasing resulting from the presentation of the social cue indicating reward availability (SDD) and its absence under the control condition (WDD). Histograms [(F) and (G)] show the distribution of chasings [habituation period (before) versus period when the stimuli carrying social information were presented (after)]. (**H** and **I**) Spectral analysis of the social network. The animals that contribute the most to the social network before the information about the reward availability is introduced are also the ones that determine changes after the social cue for the SDD was presented. The size of the horizontal bars represents each individual’s contribution (different colors) to the composition of the main factor (factor 1) underlying the shape of the network. ***P < 0.001. SDD, sucrose-drinking demonstrator; WDD, water-drinking demonstrator.

Mice are known to form complex social structures (*39*–*41*). Here, we visualize the social networks in the Eco-HAB–housed mice with a node-edge graph. In the graph, the nodes represent the number of chasings (fig. S2) performed by an individual mouse, and the thickness of the edge connecting any two nodes represents how many times a given mouse chases after another (Fig. 2, A and B). By performing simple habitation experiments—no changes introduced to the Eco-HAB environment throughout the testing period—we show that mice gradually develop a complex social network, with differences in the level of chasings between individuals stabilizing within at most 3 days of being put into the new territory (fig. S3).

Social structures in rodents are most commonly related to social hierarchy (*42*–*44*). Thus, to investigate the relationship of the social network based on the intensity of chasing conspecifics with a dominance hierarchy, we compared the number of chasings performed by the individual mice with their performance in the U-tube dominance test. We show that, in the well-stabilized social network, there is a positive correlation between the number of chasings and the U-tube dominance score (fig. S4). Notably, we show that social networks within the tested cohorts can take one of two forms: They can be hierarchical, with a clear leader (sometimes more than one), or be more egalitarian, as based on the spectral analysis showing individual contributions to the network formation. Furthermore, the effects of information about the reward availability on the social structure depend on how the network looked before changes were introduced to the testing environment.

We show that social networks change in response to the pertinent source of information being placed in the habitat. Specifically, upon the introduction of the reward availability-related social cues, the group structure shifts, in comparison to the no-change effect under the neutral social cues condition (Fig. 2, C to G). The effect is reflected in the increased frequency of chasing on the level of the whole group (Fig. 2F versus Fig. 2G). Moreover, the individual differences in the position within the stratified social network are reflected in the variable increase in chasing. This is illustrated by the spectral analysis, showing that animals that contribute the most to the social network before the information about the reward availability is introduced are also the ones that determine changes in the network to the highest degree (Fig. 2, H and I). The social networks formed by the mice in the cohort illustrated in Fig. 2 display a hierarchical structure under both tested conditions (SDD and WDD). A few individuals notably influence the social dynamics, with the dominant animal contributing the most to the observed changes. Together, social structure changes in response to social cues indicating a sucrose resource in the environment.

### The scent of a rewarded mouse attracts other mice regardless of the type of reward

To test whether interest in the social scents of familiar conspecifics exposed to the appetitive factors is evoked regardless of the type of a reward, and to ensure that the observed behavioral changes are not specifically due to the animals responding to sucrose metabolites in the social scents, we conducted the following experiments.

**Fig. 3.**
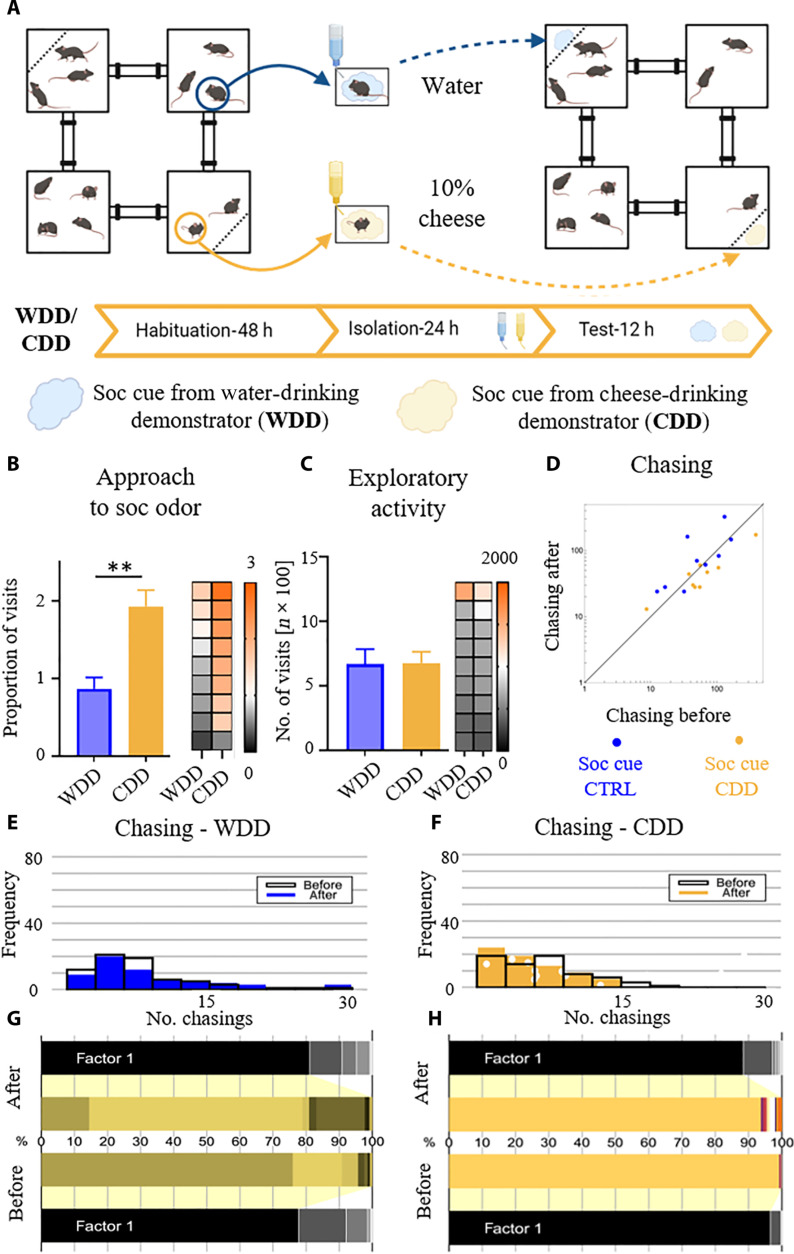
Social olfactory cues indicating a fatty reward attract mice but do not change the social dynamics. (**A**) Schematic representation of the experimental design. Arrows indicate the relocation of the animals or their scents. (**B**) Mice prefer the compartment where the bedding soaked with the scent of the CDD was presented over the compartment where the bedding soaked with the scent of the WDD was presented. (**C**) Social olfactory cue indicating that reward availability does not change exploratory activity. (**D**) The presence of the olfactory cue from a CDD does not influence chasing behavior. Histograms (**E** and **F**) show the distribution of chasings between all mice in the dark phases of the experiment [habituation period (before) versus period when the stimuli carrying social information were presented in the environment (after)]. (**G** and **H**) Spectral analysis of the social network. The animals that contribute the most to the social network structure before the information about the reward availability is introduced are also the ones that determine changes in the network to the highest degree after the social cue for the CDD was presented. The size of the horizontal bars represents individual’s contribution (different colors) to the composition of the main factor (factor 1) underlying the shape of the network. Values for individual subjects in the bar graphs are presented on the heatmap, squares in each row represent data for the same mouse, and columns represent trials, sorted by the control condition. Data in the bar graphs are shown as means ± SE. The measures in (B) and (C) were calculated as for the corresponding data in Fig. 1. **P < 0.01. CDD, cheese-drinking demonstrator; WDD, water-drinking demonstrator.

First, we conducted a test following the same protocol as presented in Fig. 1, but this time, the social scent came from a mouse that consumed a rewarding, however, non-sugary, fatty drink (Fig. 3A). As before, the mice underwent two rounds of experimental procedures.

In the control round of testing, separated mice got access to tap water (WDD). In the experimental round, one drank a 10% liquid cheddar cheese solution [cheese-drinking demonstrator (CDD)], whereas the other drank water. Similarly to what was observed in the case of the social cue indicating a sugary reward, mice enhance their interest in the bedding from the CDD in comparison to the control (Fig. 3B). Meanwhile, their exploratory activity does not change between the conditions (Fig. 3C). However, what distinguishes this experiment from the one in Fig. 1 is that exposure to the social cue from a conspecific consuming a fatty drink does not appear sufficiently potent to induce alterations in the social network. The distribution of chasing behavior does not change either in the control (WDD) or in the experimental (CDD) condition after social cues are presented in the territory (Fig. 3, D to F). Nevertheless, the structure of the group is similarly hierarchical to the one in Fig. 1, with two mice contributing the most, as indicated by the spectral analysis (Fig. 3, G and H).

**Fig. 4.**
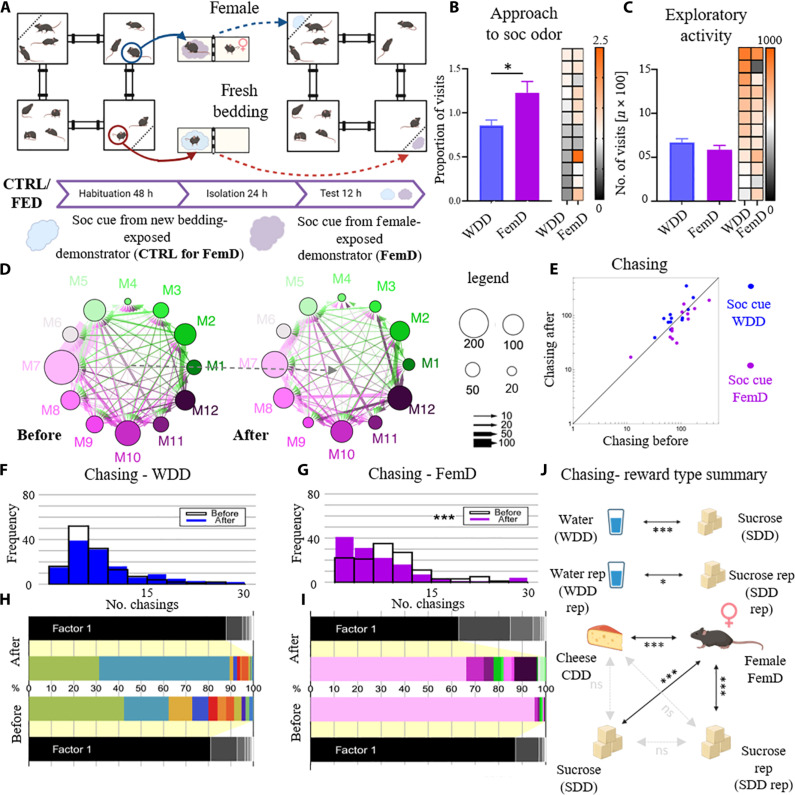
Social olfactory cues indicating exposure to a female attract mice and dampen chasing behavior. (**A**) Schematic of the experimental design. Arrows indicate the relocation of the animals or their scents. (**B**) Mice show preference for the compartment with the bedding soaked with the scent of the female-exposed demonstrator (FemD) in comparison to that of the WDD. (**C**) The FemD social olfactory cue does not influence exploratory activity. (**D** and **E**) The FemD social olfactory cue decreases chasing behavior. Histograms (**F** and **G**) show the distribution of [habituation period (before) versus period when the stimuli carrying social information were presented (after)]. (**H** and **I**) Spectral analysis of the social network. The animals that contribute the most to the social network structure before the information about the reward availability is introduced are also the ones that determine changes in the network after the social cue for the CDD was presented. The size of the horizontal bars represents individual’s contribution (different colors) to the composition of the main factor (factor 1) underlying the shape of the network. (**J**) Summary of the differences in chasing patterns in experiments from Figs. 1 to 4 and fig. S1, which differ between the conditions of exposure to the neutral and reward-indicating social cues and between the social cues indicating different types of rewards. Values for individual subjects in the bar graphs are presented on the heatmap, squares in each row represent data for the same mouse, and columns represent trials. Data are shown as means ± SE. All the measures in (B) ad (C) were calculated as for the corresponding data in Fig. 1. *P < 0.05; ***P < 0.001. FemD, female-exposed demonstrator; CTRL, new bedding-exposed demonstrator; WDD, water-drinking demonstrator.

In the next experiment, we slightly modified the original protocol to test the effects of a social cue indicating a nonconsummatory reward (Fig. 4A). Specifically, under the experimental condition, we put the separated demonstrators into the cages with dividers. One of the isolated mice was placed in a cage where, behind a divider, there was only fresh bedding (CTRL). The other demonstrator was exposed to a female separated by a divider (FemD). In this setup, the male demonstrator could not freely interact with the female or acquire her scent on its fur; it could only have nose-to-nose contact through small holes in the perforated divider. As before, we collected the scents from both the CTRL and FemD animals and presented them to the group in opposite cages of the Eco-HAB. The control condition was as previously configured in Figs. 1 and 3 (WDD). We demonstrate that mice exhibit more intense exploration of the social cue from the FemD compared to the control (Fig. 4B). As previously described, exploratory activity does not differ between the experimental and control conditions (Fig. 4C). Moreover, the presentation of such a social olfactory cue alters the level of chasing behavior within the group, thereby influencing the structure of the social network (Fig. 4, D to G). Specifically, unlike the social cues related to sucrose availability, social cues from the female exposed conspecific decrease the number of chasings, as depicted in the histograms showing changes in the distribution of this behavior within the group after the introduction of the FemD’s social cue, with no change observed under the control condition (Fig. 4, F and G). Consistent with the previous two experiments, we observe a hierarchical social order, with one or two mice contributing the most to the formation of the social network, as indicated by the spectral analysis (Fig. 4, H and I).

Together, both experiments confirm that, in the familiar environment, animals are more interested in investigating social scents indicating that the conspecific undergone an appetitive experience in comparison to the social scents of familiar mice exposed to neutral stimuli. Moreover, chasing patterns differ not only between the exposure to neutral and reward indicating social cues but also between social cues indicating different types of rewards, as depicted in the summary (Fig. 4J). It is noteworthy that chasing behavior does not vary between different experiments where we presented social cues indicating consummatory rewards. However, all the consummatory rewards chasing patterns differ from the one in condition with social cue indicating a female.

### Disrupted synaptic plasticity in the PL prevents the reshaping of social networks

**Fig. 5.**
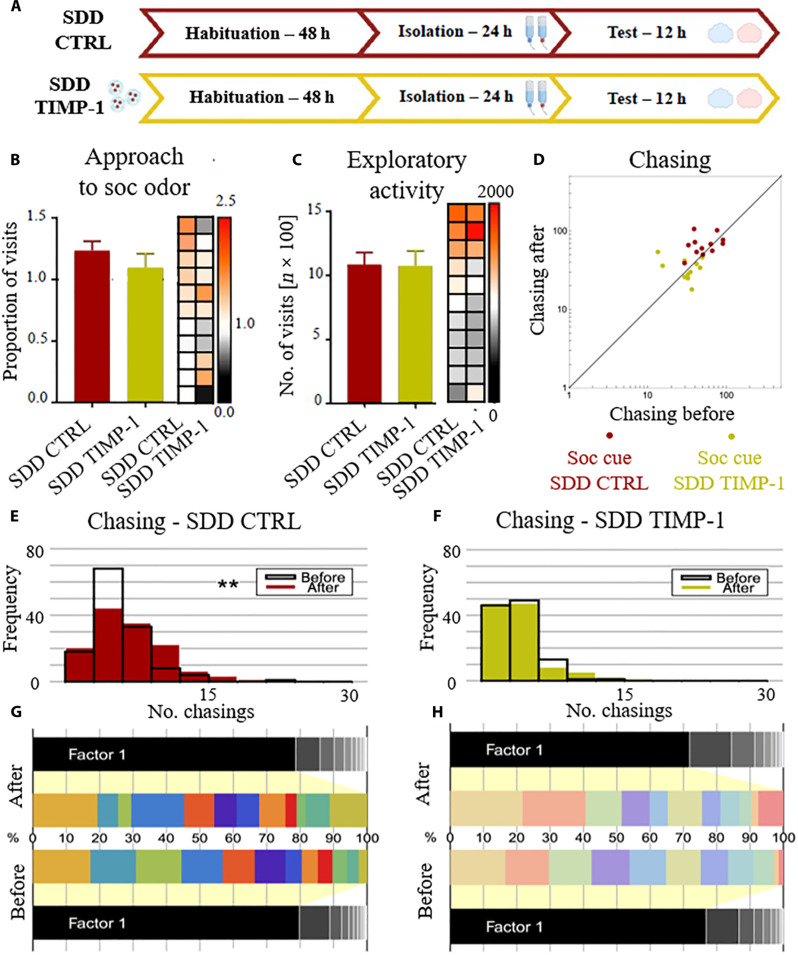
Disrupting synaptic plasticity in the PL impairs social cue–related changes in the social network. (**A**) Timeline of the experimental protocol. First, we tested the response of mice to the olfactory cues from an SDD (SDD CTRL), and then the mice were stereotaxically injected with NPs gradually releasing TIMP-1 and thus impairing neuronal plasticity in the PL. After 5 days of recovery, mice were retested in the Eco-HAB (SDD TIMP-1). (**B**) In the familiar environment, TIMP-1 injection to the PL does not change the preference for the olfactory cues from a rewarded mouse when compared to the trial before surgery, (**C**) nor does it change exploratory activity of mice. (**D** to **F**) TIMP-1 (SDD TIMP-1) disrupts the intensification in chasing between the before and after periods observed under the (SDD CTRL) condition. (**G** and **H**) Spectral analysis of the network revealed a more egalitarian (without a clear leader), however, similarly consistent group structure in comparison to the previous experiment, both before (SDD CTRL) and after TIMP-1 treatment (SDD TIMP-1). The measures in (B) and (C) were calculated as for the corresponding data in Fig. 1. **P < 0.01. Data in the bar graphs are shown as means ± SE. Values for individual subjects are presented on the heatmap (right to the bar plots), squares in each row represent data for the same mouse, and columns represent trials, sorted by the control condition.

In the next experiment, we used the previously established protocol (Fig. 1A), exposing a demonstrator to a sucrose reward, to study the behavior of mice before (SDD CTRL) and after impairing their neuronal plasticity by releasing TIMP-1 into the PL (SDD TIMP-1, Fig. 5A). Initially, we tested a cohort of naïve mice by presenting them with social cues from SDDs in the familiar environment, as done with the SDD group in Fig. 1. Following this, we bilaterally injected NPs loaded with TIMP-1 to the PL, which markedly altered the observed behavioral pattern.

Notably, after the brain manipulation impairing neuronal plasticity in the PL (SDD TIMP-1), mice still display preferences for the social scent indicating a reward similar to the control condition (SDD CTRL, Fig. 5B). Also, the injection of TIMP-1–loaded NPs does not affect the overall exploratory activity (Fig. 5C). However, this brain manipulation eliminates the changes in the social network induced by social cues indicating a sucrose reward, seen under the SDD CTRL condition (Fig. 5, D to F). The social network, characterized by a more egalitarian structure within this group and the lack of clear leaders, remained unchanged even after the introduction of social information regarding reward availability, across all experimental conditions. This is supported by spectral analysis (Fig. 5, G and H) indicating the stability of the social network within a given group over time. Thus, disrupting neuronal plasticity in the PL prevents the usual reshaping of social network induced by social cues indicating a sucrose reward presented in the familiar environment.

### Social olfactory information helps to navigate a novel environment

**Fig. 6.**
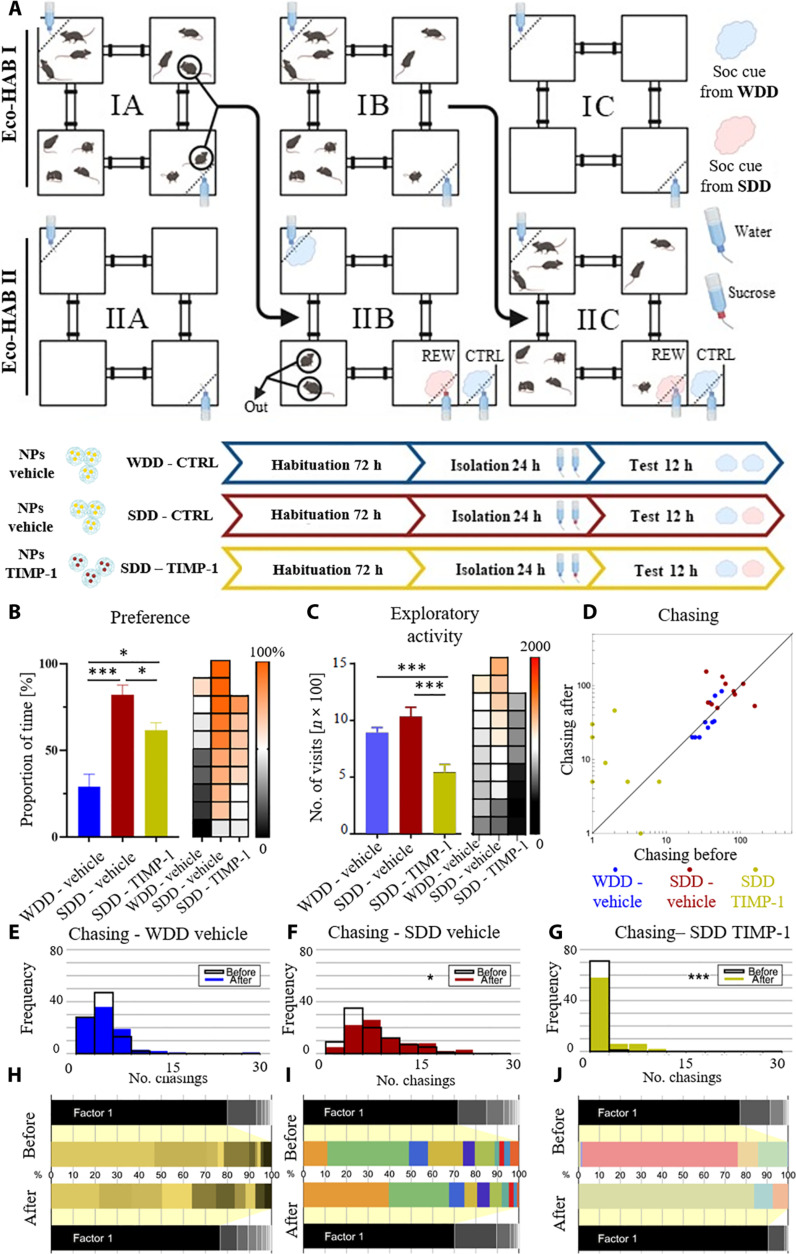
Social olfactory information helps to find the reward in a novel environment; the process requires intact plasticity in the PL. (**A**) Schematic of the experiment. During habituation (IA and IIA), we placed additional water bottles in both Eco-HABs. After 72 hours (IA), two mice from Eco-HAB I were moved to the intact Eco-HAB II (IIB), leaving the rest of the group behind (IB). One bottle in the novel Eco-HAB II contained 10% sucrose, the other tap water (SDD-vehicle); in the control group, both bottles contained water (WDD-vehicle). After 24 hours, the two scouts were moved out, the bottles cleaned and refilled with water, and the rest of the group was moved into the Eco-HAB II (IIC). (**B**) Newcomer mice prefer drinking from the bottle in the compartment where the scout mice were rewarded (WDD-vehicle versus SDD-vehicle). Injection of TIMP-1 decreases that preference (SDD–TIMP-1). (**C**) The exploratory activity is unchanged between the WDD-CTRL and SDD-CTRL conditions. Meanwhile, SDD–TIMP-1 mice show a decrease in this parameter in comparison with both those groups. (**D** to **G**) In the SDD-CTRL and SDD–TIMP-1, chasing intensifies upon introduction to the new environment, which is not the case in the WDD-CTRL. However, the overall chasing is diminished in the SDD TIMP-1 group. (**H** to **J**) Spectral analysis of the social networks in WDD-CTRL and SDD-CTRL groups reveals that they are stratified and have clear leaders. The animals that contribute the most to the composition of the social network before the change of the habitat are the ones that contribute the most after. In the SDD–TIMP-1 mice, the structure of the social network is unstable and scrambled. *P < 0.05; ***P < 0.001. Data in the bar graphs are means ± SE. Values for individuals in (B) to (C) are presented on the heatmap in accordance with the order of bars and sorted.

To investigate whether olfactory information from a rewarded mouse helps to find resources in a novel, unfamiliar environment, we tested mice transferred to a new Eco-HAB system, previously inhabited by two familiar mice (scouts), which had left social olfactory cues throughout the territory (Fig. 6A). These social cues had been left by the animals that had access to either water in the two opposite Eco-HAB compartments (WDD-vehicle) or to a 10% sucrose solution in one of the compartments and to water in the other compartment (SDD-vehicle and SDD–TIMP-1). The bottles drunk from by the scouts were replaced with the new ones containing water before the tested cohort of mice—moving in from another Eco-HAB—was introduced to the testing environment.

We show that mice in the SDD-vehicle group display a preference for the bottle placed in the site previously rewarded in scouts (Fig. 6B), whereas their exploratory activity remains unchanged (Fig. 6C). These results were confirmed by the replication experiment performed in another cohort of mice (fig. S5). In addition, mirroring previous experiments, mice exhibit changes in chasing behavior upon exposure to social cues indicating reward availability in the environment, contrasting with the control condition (Fig. 6, D to F). Notably, our replication experiment (fig. S4) supports these observations.

On the other hand, mice with TIMP-1–impaired neuronal plasticity in the PL (SDD–TIMP-1) exhibit deficits across all tested behaviors. Specifically, they display a reduced preference for the site where the scouts were rewarded (Fig. 6B) and engage in much less chasing behavior (Fig. 6, D and G) even if it is slightly increased after presentation of the social cue indicating sucrose. Furthermore, all cohorts demonstrate a clear hierarchical social structure, as evidenced by spectral analysis highlighting a few animals that are contributing the most to the chasing patterns (Fig. 6, H to J). However, in contrast to the nontreated groups (Fig. 6, H and I), the TIMP-1–treated animals show disrupted contributions to the composition of their social network. Namely, those mice that were the most notable contributors prior to the social cues presentation are not the same individuals that are the most notable contributors afterward (Fig. 6J). Nevertheless, this result should be interpreted cautiously as the level of chasing in the group is notably lower compared to the nontreated animals tested under the SDD-vehicle condition (Fig. 6D). In addition, the SDD–TIMP-1 group displays a slight decrease in exploratory activity (Fig. 6C). Therefore, these data underscore that, under the more challenging conditions of a new environment, deficits in neuronal plasticity in the PL not only affect social behavior but also influence exploratory activity.

### Acute silencing of the PL impairs the response to socially cued reward but not chasing

Under the challenging conditions of a novel environment, prolonged TIMP-1 treatment in the PL affects both the exploration of social cues signaling reward availability and the patterns of pursuit within social networks (chasing). To discern whether these effects stem specifically from dysfunctional neuronal plasticity rather than acute impairment of neuronal activity, we conducted chemogenetic experiments.

**Fig. 7.**
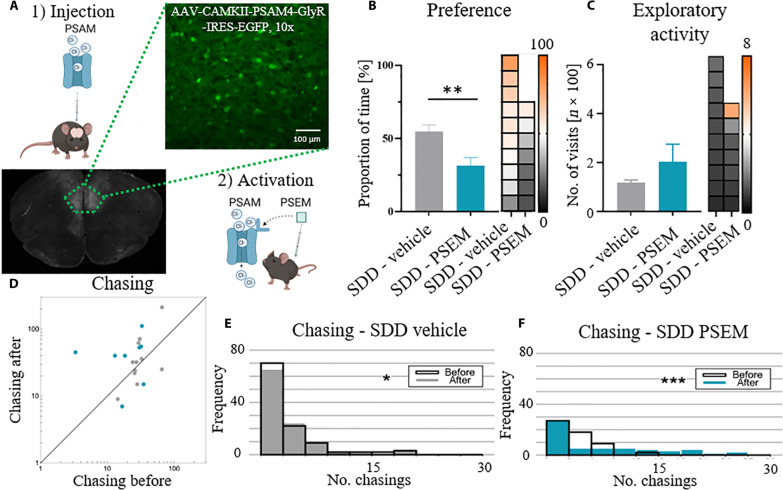
Silencing of the excitatory CaMKII-expressing cells in the PL impairs social learning but does not prevent the changes in chasing behavior. (**A**) We silenced excitatory cells in the PL with ultrapotent chemogenetics (PSAM-PSEM system). Animals were injected with a vector expressing PSAM under the CaMKII promoter in the PL and subjected to the novel environment Eco-HAB experiment after 3 weeks. The artificial chloride ion channels were activated with an intraperitoneal injection of PSEM for ~120 min when animals were put into the new environment with social cues indicating a reward. (**B**) Mice with silenced PL neurons (SDD-PSEM) display a lower level of preference for drinking from the bottle in the compartment where the scouts were rewarded in comparison to the control, saline-injected group (SDD-vehicle). (**C**) Exploratory activity is not affected by the brain manipulation. (**D** to **F**) Longitudinal patterns of chasing behavior measured after the chemogenetic intervention has ceased (6 to 12 hours after PSEM injection). The frequency of chasing increased upon the presentation of the social cues indicating a sucrose reward under both SDD vehicle and SDD PSEM conditions in comparison to the baseline period, showing that acute reduction in neuronal activity in the PL has no impact on reshaping of social networks. *P < 0.05; **P < 0.01; ***P < 0.001. Data in the bar graphs are shown as means ± SE. Values for individual subjects in (B) and (C) are presented on the heatmap (right to the bar plots) in accordance with the order of bars; each group is sorted.

We used an ultrapotent pharmacologically selective actuator module (PSAM)–pharmacologically selective effector molecule (PSEM) system (Fig. 7A), which allows to alter neuronal activity for at least 120 min (*45*). We silenced the excitatory, calcium- and calmodulin-dependent protein kinase II (CaMKII)–expressing neurons in the PL and performed the studies on mice using social information about the reward to navigate in a novel environment, as previously described (Fig. 6A). We measured the effects of the intervention both during the neuronal silencing and after it has ceased to see whether the longitudinal effects on reshaping of social network present in the TIMP-1–treated animals are also present in this case.

We demonstrate that silencing excitatory neurons in the PL (SDD-PSEM) selectively impairs interest in social olfactory cues indicating a reward. This is evidenced by the reduced preference for the bottle rewarded in scout mice compared to the control group (SDD-vehicle, animals injected with saline instead of the PSEM, Fig. 7B). There are no differences in the exploratory activity between the groups (Fig. 7C). However, in contrast to the plasticity-related effects of TIMP-1, neuronal silencing does not prevent reshaping of the chasing patterns caused by the presentation of social cue indicating a sucrose reward in the long run. To test that, we calculated the frequency of chasing under both the SDD-vehicle and SDD-PSEM conditions in comparison to the baseline period after the chemogenetic intervention has ceased (6 to 12 hours after PSEM injection). To evaluate the direct impact of the chemogenetic intervention, we also looked at chasing in the 2-hour period of neuronal manipulation (right after PSEM injection), but as expected, such a short time is not sufficient for animals to show any changes in social network (fig. S7, A to C). The analysis of the broader, 6-hour period after the injections reveals that the reshaping of chasing patterns start appearing in the SDD-vehicle group already at this point but not yet in the PSEM-manipulated animals (fig. S7, D to F), which are then explicit after 6 to 12 hours after the intraperitoneal injections (Fig. 7F).

Thus, although deficits of the synaptic plasticity disrupt chasing patterns within the social network (Fig. 6, D, G, and J), short-term changes in neuronal activity do not affect this behavior. Together, our findings suggest that intact neuronal plasticity in the PL is crucial for maintaining social structure within the group, as observed in our experiments conducted in the novel Eco-HAB environment (Figs. 6 and 7).

## DISCUSSION

We show that mice eagerly explore social olfactory cues left by their rewarded conspecifics and can distinguish between neutral and reward-related social odors, regardless of the reward type. Furthermore, the olfactory information left by a rewarded mouse helps other individuals navigate in a novel environment. In addition, we show that olfactory cues from well-known, familiar conspecifics suffice for the transfer of information about the reward. This observation is in line with the social information transmission theory, according to which, in a novel environment, animals with no direct experience heavily rely on social cues (*46*). Consistently, we observed that, after the two scout mice had been patrolling and scent marking the novel environment, the rest of the mice, subsequently moved into the Eco-HAB, preferred drinking from the bottles that were rewarding for their peers. The effects of gaining preference for the things liked by the members of one’s social group are well documented in humans (*47*). The presented experimental approach opens previously unexplored avenues for investigation of such phenomena and enables a better understanding of their neural basis in well-controlled rodent experiments.

Notably, a critical aspect of this process is the ability of mice to distinguish and respond differently to the scents of familiar conspecifics. Rodents, in particular, are known to recognize the social scents of individuals they have been housed with (*15*, *48*–*53*), regardless of genetic relatedness (*54*). These scents are thought to consist of unique signature mixtures, rather than simple pheromones (*51*, *52*, *55*–*59*). The complex volatile profiles each mouse produces play a critical role in their ability to recognize others. Notably, a change in the scent of a familiar individual—potentially induced by exposure to a new environment, such as a novel cage—often prompts close examination by other mice (*54*). Given this, the influence of a social scent from an animal that drank water cannot be disregarded. To account for the potential impact of novelty, we simultaneously presented subjects with two familiar social scents: one from an animal that drank water and another from an animal that consumed sucrose and cheese or had been exposed to a female. The same protocol was applied under the control conditions, where the social scents were from two different familiar animals exposed to neutral stimuli. Despite both demonstrators encountering novelty, the test subjects reliably alter their behavior in response to the scent of the animal subjected to the experimental condition, even in cases where metabolic changes are unlikely to play a role, such as exposure to a female. Thus, the differential responses of the test subjects suggest that the behavioral shifts are driven primarily by the demonstrator’s experience, conveyed through olfactory cues released by the demonstrator, rather than by metabolic by-products or general novelty.

Furthermore, social learning relies on efficient social communication (*60*) and social information processing (*61*). We have developed a method of tracking social interactions between individual mice living in a group in the Eco-HAB by tracing chasings through the corridors of the system. We chose to measure chasing because it is a type of social interaction by definition voluntarily initiated by a mouse, which chooses to trail another and thus gets direct access to the olfactory cues of a trailed individual. Getting olfactory cues through sniffing the anogenital area of other individuals is an important source of information in mice (*62*). Because changes in chasing behavior show high individual variability, they may play a role in social communication. Moreover, except for information seeking, chasing is also a form of dominant behavior (*63*–*68*). Notably, the presence of social sucrose reward–related olfactory cues in the environment increases chasing. The degree to which chasing increases reflects individual differences in social status within the group. Thus, it is likely that the chasing behavior has two components: information seeking and expression of dominance over other mice (*65*, *66*).

To have an independent measure of social dominance, we compared the level of chasing of individual mice with dominance status determined by the U-tube test and found a positive correlation between the two measures. However, we acknowledge that the latter may be considered a suboptimal method for assessing dominance hierarchy as it forces animals to compete in a constrained environment. Studies have shown that tube test results have weak correlation with actual dominance as measured by agonistic interactions such as fighting (*69*, *70*). In addition, in the spacious and variable environment of the Eco-HAB, animals rarely exhibit overt aggression due to the ample space available for individuals to hide or avoid one another. Consequently, such interactions are infrequent. Therefore, incorporating other reliable measures of dominance, such as territoriality, would be beneficial in future studies to fully understand the role of chasing behavior in the Eco-HAB.

We used spectral analysis of the chasing data to identify the animals that contribute the most to the formation of the social network. Although we are aware of the existence of other measures of this property, such as the steepness coefficient based on David’s scores, we find it unsuitable for the analyzed behavior of mice. We argue that this may be because the chasings are not aggressive behavior (as opposed to direct fights that end with a clear win or loss) and their functional meaning can be manifold, with information passing as a more fundamental function. We also note that the steepness coefficient is quite insensitive to changes in the social network detectable by other means (figs. S7 to S11).

It is noteworthy that the presented social network distribution in mice tested in Eco-HAB shows similarities with social networks observed in human research (*71*). Tracking individual differences in the positions occupied within the social network can be very useful for increasing our understanding of associated behaviors, as well as for modeling their impairments (*72*). Here, we show how to analyze them with the use of seminaturalistic experiments performed in the Eco-HAB system. The term “seminaturalistic” refers to the fact that the testing environment was designed to mimic crucial characteristics of the murine natural habitats (*38*, *73*). Specifically, it consists of multiple burrow-imitating cages, each with two corridors leading in and out, as found in the field studies of rodents (*74*–*76*). In addition, the mice are kept in groups but are not subjected to the presence of individuals they do not wish to interact with; thus, the setup allows for the formation of voluntary relationships between the animals. Moreover, the design of the environment permits substantial social and exploratory activity, as the mice can visit different parts of the enclosure, and allows for comparable variability in territory occupation compared to standard behavioral tests or housing cages available in most animal facilities. Although this setup is not identical to natural conditions, we argue that its design effectively mimics key characteristics of natural habitats, enabling the mice to exhibit species-typical behaviors. In most of the classical tests of sociability, randomly chosen pairs of mice are tested, which can increase variability of the results and blur the conclusions. In contrast, longitudinal observation of mice in Eco-HAB enables tracking of complex, voluntary, and dynamic interactions, thus far exceeding the limits of what can be studied in the conventional approaches to measuring social behavior.

A notable limitation of our studies is the variability observed at both the individual and group levels. Experimental murine social systems exhibit differences within and across treatments; some cohorts display more despotic behaviors, whereas others are more egalitarian. This variability complicates efforts to investigate the consistency of observed effects, similar to challenges faced in field studies. However, we posit that this natural variability presents a valuable opportunity to study these phenomena directly, which are often overlooked in traditional behavioral experiments. Our study is among the few that uses an automated methodology to focus on individual variability as a central research topic, rather than treating it merely as a confounding factor in group data analyses.

Moreover, even when selecting groups of mice that share the same genotype and come from the same colony, we find substantial differences in their social structures. Individual mice also exhibit varied responses to environmental changes. Consequently, our study emphasizes the individual behavioral changes that arise when mice are exposed to the scent of a familiar conspecific that has experienced rewarding stimuli (whether food-related or otherwise) within a complex social context. Although this complexity poses challenges for interpretation, it effectively mirrors real-world conditions. Furthermore, our findings suggest that genetic similarity alone does not account for most of the variability in group structures. We acknowledge, however, that studies examining individual variability necessitate extensive replication to ensure scientific rigor. Further investigations are essential to fully unravel the complexities of the behaviors discussed and the intricacies of the social systems formed by mice.

The PFC is essential for successful navigation through a complex social world, both in humans and in other social species, especially the dorsomedial PFC and medial PFC have been implicated in the monitoring of emotions and actions in self and others (*23*, *77*–*81*). The functional homolog of these regions in rodents is the dorsomedial PFC, including the anterior cingulate cortex and the PL (*24*).

We show that disrupting neuroplasticity in the PL with TIMP-1 results in reduced response to social olfactory cues indicating a reward, under novel conditions. This is in line with the earlier study showing that the PL neurons selectively respond to social olfactory cues (*48*). Neuronal plasticity in this structure has been also shown indispensable for the maintenance of social structure (*24*, *65*, *82*). The NPs used in the experiments gradually released TIMP-1 over several days (*83*), leading to a prolonged impairment in the updating of neuronal connectivity in the PL. We show that such a prolonged, TIMP-1–induced impairment of neuronal plasticity in the PL results in not only markedly diminished interest in seeking social reward–related olfactory cues in novel environment but also disrupted patterns of chasing after other animals in the presence of such cues in both familiar and novel environments. In contrast, brief chemogenetic silencing of the PL only leads to the former. This result points to the conclusion that the ongoing adjustment of the neuronal connectivity within the PL might be critical for maintaining social structure. This is also in line with the recent rodent studies, which have shown that learning and experience-dependent synaptic plasticity in the PFC are critical for social rank status (*24*, *61*). Notably, impairing neuronal plasticity in the PL strongly decreased the overall number of chasings performed in the brain-manipulated groups even before the social cues indicating a reward were presented in the environment. Furthermore, impairing the plasticity disrupted how the structure of the social network changes in response to information about a sucrose reward being present in the environment. However, the considerable general decrease in chasing in these experimental groups makes this result hard to interpret unequivocally.

The presented behavioral approach can be further used to study mechanisms of social learning, in particular social learning strategies (*60*). Reward learning, in contrast to threat learning, is more dependent on when and from whom one learns to optimize behavior (*61*, *84*). As searching for a reward is an investment, being able to estimate the effort needed to get it and the attractiveness of the bounty are important. Thus, the reliability and accessibility of the source of knowledge is key.

## MATERIALS AND METHODS

### Subjects

Animals were treated according to the ethical standards of the European Union (directive no. 2010/63/UE) and respective Polish regulations. All the experiments were preapproved by the Local Ethics Committee no. 1 in Warsaw, Poland, ethics permit no. 740/2018 with the following extensions. C57BL/6 male mice were bred in the Animal House of Nencki Institute of Experimental Biology, Polish Academy of Sciences, or Mossakowski Medical Research Centre, Polish Academy of Sciences. Because of the large groups of mice required for the behavioral tests presented in this manuscript (12 to 15 for each experimental condition), we conducted the experiments using only one sex. Whenever possible, the experiments were conducted in a way enabling within subject comparisons. These decisions were made to comply with the 3R (Replacement, Reduction, and Refinement) guidelines and recommendations of the local ethics committee, which aim to minimize animal use in research. We acknowledge that this approach may limit the extrapolation of the results to female subjects. Therefore, further research is necessary to explore potential sex-specific differences and validate these findings more comprehensively. Experiments were performed in 12 cohorts of mice, with each main experiment being replicated twice. The animals entered the experiments when 2 to 3 months old. They were littermates derived from several breeding pairs. The mice were transferred to the animal room at least 2 weeks before the experiments started and put in groups of 12 to 15 in one cage (56 cm by 34 cm by 20 cm) with an enriched environment (tubes, shelters, ad nesting materials). The information on *n* of animals used and how many cohorts of animals per cages were used in the particular experiments can be found in the experiment’s description and table S1. Reported *n* is sometimes smaller than 12 to 15 due to the animals with nonqualified bilateral injections removed from the analysis and demonstrators/scouts permanently removed from the cohorts; for details, please see the experimental protocols below. Mice were kept under a 12-hour/12-hour light/dark cycle. The cages were cleaned once per week.

### RFID tagging

Glass-coated radio frequency identification (RFID) microtransponders (9.5 mm in length and 2.2 mm in diameter, RFIP Ltd.) were sterilized in 70% ethanol, dried on a paper towel, loaded into the syringes, and injected subcutaneously into the subjects anesthetized with isoflurane. Each transponder had a unique number that could be registered by the Eco-HAB antennas when animals pass through its corridors. After injections, mice were put together into one home cage and moved back to the experimental room. On the next day, the presence and the position of the transporters under the animals’ skin were additionally verified.

### Poly(dl-lactide-*co*-glycolide) NPs containing TIMP-1 or BSA

To gradually release TIMP-1 in the PL of the tested animals, we used poly(dl-lactide-*co*-glycolide) (PLGA) NPs loaded with the protein [under the control condition bovine serum albumin (BSA), Sigma-Aldrich]. The NPs were prepared according to the protocol described by Chaturvedi *et al.* (*83*). Briefly, NPs were prepared in the process of multiple emulsifications and evaporations [molecular weight (MW) 45,000 to 75,000, copolymer ratio: 50:50, Sigma-Aldrich]. One hundred milligrams of PLGA was dissolved in 5 ml of dichloromethane and 4 ml of dimethyl tartaric acid (Sigma-Aldrich). In the next step, 1 mg of TIMP-1 or BSA was dissolved in 500 μl of Milli-Q water. The protein solution was mixed with dichloromethane containing PLGA, sonicated, and emulsified in 1% polyvinyl alcohol (on average, MW 30,000 to 70,000, Sigma-Aldrich). In addition, fluorescein isothiocyanate (FITC) was added to easily localize the place of NP’s delivery in the brain. Subsequently, the solution was stirred at room temperature overnight to evaporate dichloromethane. Next, the NPs were centrifuged at 10,000*g*, washed three times with Milli-Q, dissolved in phosphate-buffered saline (PBS), and stored at 4°C.

### Chemogenetics

We used the chemogenetic system using PSAMs engaged by engineered agonists and PSEMs, which provides high substrate-ligand selectivity while keeping the functional effects typical for the ion channel to which the PSAMs are combined (*45*, *85*, *86*). We induced the CAMKII-dependent expression of the PSAM in the pyramidal cells of the PL via the inhibitory construct AAV-CamKII-PSAM4-GlyR-IRES-EGFP (Addgene). The viral vector was injected into the animals’ brains bilaterally (250 to 300 nl per site) before the start of the experimental procedures. To manipulate neuronal activity in situ, mice that were kept in the Eco-HAB were briefly taken out of the territory and intraperitoneally injected with the PSEM solution (uPSEM 792, 1 mg/kg body mass, in saline, Tocris). Next, animals were placed back into the Eco-HAB and subjected to testing of spontaneous behaviors, as described in the following sections. After the experiments, the injection sites were verified, and only the animals that were correctly bilaterally injected into the PL were included in the analysis.

### Stereotaxic surgeries

All tools were sterilized in 70% ethanol before the surgical procedures. Mice were anesthetized by isoflurane inhalation (started at 5% and reduced to 2 to 1.5% of isoflurane) and placed in a stereotaxic apparatus (Kopf Instruments) on a heating pad (37.8°C). The mice were subcutaneously injected with an analgesic (Butomidor, Richter, 1:20 in saline, 2.5 ml/kg), and reflexes were checked to ensure the absence of pain. To protect the animals’ eyes from drying, we used a moisturizing gel (Carbomerum, Vidisic). Ear bars were put into place, and the scalp was shaved. The skin on the skull was cut to expose bregma. NanoFil 35-gauge needles were used to bilaterally inject NPs into the PL [coordinates: anterior-posterior (AP), +1.8 mm; lateral-medial (LM), ±0.92 mm; and dorsal-ventral (DV), −1.67 mm; at a 20° angle]. The delivery was controlled by the Micro Syringe Pump (World Precision Instruments, 500 nl of total volume, 100 nl/min). To let the NPs diffuse, the needle was left in the brain for an additional 5 min after the injection. Afterward, the incision was sutured (Dafilon, C0935204) and lubricated with the analgesic lignocainum hydrochloricum (10 mg, Polfa). The mice received subcutaneous injections of anti-inflammatory medication (Tolfedine, Vetoquinol, 4 mg/kg) and a wide-spectrum antibiotic (Baytril 2.5%, Bayer, 1:3 in saline, 5 ml/kg). Then, mice were placed in cages warmed with a heating pad and singly housed for the next 5 days to allow for full recovery.

### Perfusions and verification of TIMP-1 and PSAM injections

After the end of behavioral testing, mice injected with the NPs releasing TIMP-1 or BSA, as well as the PSAM-injected animals, were anesthetized with an intraperitoneal injection of sodium pentobarbital (100 mg/kg, dissolved in PBS) and transcardially perfused with 80 ml of ice-cold PBS followed by 60 ml of 4% paraformaldehyde (PFA) in PBS (4°C). The brains were isolated and placed overnight in 4% PFA in PBS (4°C). Then, the brains were moved to a 30% sucrose solution in PBS for 2 to 3 days (4°C) for cryoprotection. Afterward, the brains were cut on a cryostat into 50-μm-thick coronal slices. The slices were then washed in PBS and placed on the microscope glass slides, and the fluorescence of FITC encapsulated in NPs or enhanced green fluorescent protein (EGFP) expressed in the PSAM-positive neurons was imaged under the Nikon Eclipse Ni microscope.

### Eco-HAB system

The Eco-HAB is a fully automated, open-source system for testing social behavior in group-housed mice living under the 12-hour/12-hour dark/light cycle (*38*). The system comprises four polycarbonate compartments (30 cm by 30 cm by 18 cm) connected with tube-shaped corridors (inner diameter of 36 mm and outer diameter of 40 mm) and covered with a stainless steel grid lid. In two of four compartments, mice have access to food and water (ad libitum); the other two compartments have a separate space for presentation of the olfactory stimuli (in a corner, behind a perforated partition) or putting additional bottles. Access to all compartments, olfactory stimuli, and additional bottles is unrestricted and voluntary. All housing elements can be autoclaved and disinfected with 70% alcohol. To record the movement of the animals within the Eco-HAB, the corridors are equipped with circular RFID antennas registering transponders’ numbers. The data from the antennas are collected by a dedicated electronic system, which sends them to the computer. Eco-HAB measures were computed with the custom scripts of the pyEcoHAB library. The following behavioral measures were analyzed. In a familiar environment, approach to social odor was measured as a proportion of visits to the compartments with olfactory stimuli (reward:neutral or neutral:neutral, depending on the experiment) and normalized by dividing by the same proportion from the preceding dark phase (when no olfactory stimuli were present in the compartments). In the experiments performed in a novel environment, preference for the bottle (reward:neutral or neutral:neutral) in which scout mice were presented with a reward was measured as a ratio of the time spent drinking in the compartments with olfactory stimuli during the first hour of the experiment (reward:neutral or neutral:neutral). In the chemogenetic experiments, the preference for the bottle was measured throughout the neural manipulation period, that is, 120 min after the intraperitoneal injection of the PSEM. Chasings were measured based on the number of events when mice chased after one another through the corridors of the Eco-HAB system. Specifically, chasing was defined as an event when one mouse entered a corridor (activation of the entrance antenna by mouse A), followed by another mouse (activation of the entrance antenna by mouse B) before the first left the tube (activation of the exit antenna by mouse A and subsequently by mouse B), and when both mice left the corridor in the same order and in the same direction. Notably, the behavior was not categorized as chasing when the two animals backed up to the cage they originally came from or passed one another in the corridor. The exact order of antenna activations required for the behavior to meet the definition of chasing is illustrated in fig. S2. For the analysis of within-cohort changes in chasing patterns, raw values were used.

### Testing response to social olfactory cues indicating a reward in a familiar environment

The mice were put into the Eco-HAB system at the beginning of the dark phase. Animals were habituated to the testing environment for 48 to 72 hours (habituation phase). Then, two mice randomly chosen without regard to their social status, which was neither known nor investigated at the time, were housed in the individual cages (17 cm by 23 cm by 13 cm) for the next 24 hours (isolation phase). They were offered either a neutral novel stimulus or rewarding novel stimulus. Namely, consummatory stimuli included (i) tap water or a 10% sucrose solution (Sigma-Aldrich) and (ii) tap water or a 10% liquid cheddar cheese solution (Kong Easy Treat). In a nonfood stimuli variant of the experiment, we used presentation of neutral, fresh bedding behind the perforated partition or presentation of a female (in proestrus) behind the perforated partition. Food was freely available throughout this period. Fist-sized portions of bedding soiled with urine and fecal matter from the separated mice were used as social olfactory cues. For the next 12 hours, it was put behind the perforated partitions in the Eco-HAB system to avoid spreading it throughout the cages while maintaining unrestricted sniffing. The separated mice were never put back into the original cohorts to avoid potential impact on social networks and confounding the conclusions regarding the presentation of social scents. Each cohort was tested twice; in the first trial, both separated mice had access to tap water, and in the second trial, the same mice were isolated and one of them had access to a reward while the neutral stimulus. The experiment with a 10% sucrose solution was repeated twice in two independent cohorts of mice. The cohort used for the replication was then rehoused in the animal facility for 2 weeks and then reused for the experiment with female exposure. Additional cohort was used for the fatty reward experiment. The design of the experiment with TIMP-1 released into the PL was performed following the same protocol. Similarly, the mice were tested twice: before and after brain manipulation (5 days after NP injection). As previously described, in both trials, the same pair of mice was isolated to produce social olfactory cues. Please note that, in experiments presented in Fig. 1, one mouse had to be excluded from the experiments due to health problems with fore-tooth overgrowth that might have affected its social behavior.

### Testing response to social olfactory cues indicating a reward in a novel environment

The main experiment was repeated twice (WDD versus SDD conditions). We tested the following cohorts of mice: WDD-vehicle (one mouse had to be excluded from the analysis because it had not drunk water), SDD-vehicle, SDD–TIMP-1 (two mice died after the surgeries), and WDD rep/SDD rep (within subject condition, as described with the previous experiments). Within subject design was not feasible in the brain manipulation experiment, due to the need of performing vehicle control. The mice were injected with NPs containing TIMP-1 or BSA (vehicle) 5 days before the start of the experimental procedures. In behavioral testing, two opposite Eco-HAB compartments offered access to the additional bottles through a short (8 cm long) tube, equipped with an RFID antenna to register the drinking time of each mouse. As in the previous experiments, the mice were put into the Eco-HAB system (Eco-HAB I) at the beginning of the dark phase. They were habituating to the environment for the next 72 hours (habituation phase). Then, two mice randomly chosen without regard to their social status, which was neither known nor investigated at that time, were moved into a new, clean Eco-HAB system (Eco-HAB II), in which the only bottles accessible were the ones with the antennas monitoring the drinking behavior. In the reward (SDD) trials, the scout mice had access to tap water in one compartment and to a 10% sucrose solution in the opposite compartment of the Eco-HAB II. In the control (WDD) trials, both bottles were filled with tap water. After 24 hours of the Isolation phase, the two scout mice were removed, and the bottles were replaced with the ones containing fresh tap water. The scouts were never put back into the original cohorts to avoid potential impact on social networks and confounding the conclusions regarding the presentation of social scents. Then, the rest of the group, which until now had inhabited the original Eco-HAB I, was transferred to Eco-HAB II for 12 hours (testing phase).

### Longitudinal observation of social structure formation in the Eco-HAB

To observe how social structure is formed, we tested the voluntary behavior of mice in the simple housing experiments, in which animals inhabited Eco-HAB, without any additional changes to the testing environment. Two cohorts of mice (each *n* = 12) were placed into the Eco-HAB system, and their behavior was measured for 4 days.

### Dominance tests

To assess the relationship between social structure and dominance hierarchy, we tested the subjects from one of the cohorts form the longitudinal observation of social structure formation in the Eco-HAB and U-tube tests. Following the observation of the social structure in the Eco-HAB system, the mice were subjected to the U-tube dominance test. Mice were placed in single cages and tested in all possible pairwise combinations. The mice from the currently tested pair were placed at the opposite ends of the U-shaped tube (1 m in length and 42 mm in diameter) and allowed to interact. When one mouse pushed the other out, it won a given bout. We tested all pairs and calculated the number of wins for each subject as a measure of dominance.

### Social network analysis

To analyze the social network formed by a given cohort of mice, we assessed the number of chasings for each pair of mice, i.e., the number of chasings of mouse A by mouse B for A ≠ B. In a cohort consisting of *N* mice, we thus obtained an *N*(*N* − 1)–element vector of chasings. Distributions of the number of chasings within pairs of mice under a given condition were analyzed using the Kolmogorov-Smirnov test. In the dot plots showing the chasing behavior before and after the social stimuli presentation for each mouse (log scale), data points with undefined log values are not shown. To identify subsets of mice that give dominant and independent contributions to the chasing pattern, we used singular value theorem. To this end, spectral analysis was performed on the chasing matrix X, whose entries are defined in the following way: X_AB_ is equal to the number of chasings of mouse A by mouse B for A ≠ B and X_AB_ = 0 for A = B. We calculated the eigenvalues and eigenvectors of the matrix X^T^X. The square root of a given eigenvalue corresponds to the number of chasings performed within an independent subset of mice and the squares of the components of the corresponding eigenvectors give relative contributions of respective mice to this eigenvalue. In particular, if one of the components of such an eigenvector is much larger than the other, then we conclude that a single mouse corresponding to this component is primarily responsible for an independent contribution to chasings. The comparison of the steepness coefficients was performed by calculating the right-tailed *P* value for the larger measured steepness value in *N* = 2000 synthetic random win-loss matrices with an additional condition that their steepness is at least equal to the smaller measured steepness. The randomization procedure is described by de Vries *et al.* (*87*). This part of the analysis was performed in Wolfram Mathematica.

### Statistical analyses

For statistical analyses, the normality of data distributions was assessed with the D’Agostino-Pearson omnibus normality test. Datasets that passed the normality tests were further analyzed using Student’s *t* test for independent or paired samples, depending on the particular type of comparison. For the datasets that did not pass the criterion of normality required for performing parametric analyses, the Mann-Whitney or Wilcoxon matched-pairs signed-rank tests were used. The correlation was calculated by the Pearson correlation test. To compare the distributions of chasing behavior across different experimental conditions, we used the Kolmogorov-Smirnov test to analyze the distributions of values within two respective vectors. The criterion for statistical significance was a probability level of *P* < 0.05. This part of the analysis was performed with the use of the GraphPad Prism 10.2.3 software and Wolfram Mathematica. Statistical details for all experiments are described in table S1.
