# Supplementary material for: Information sharing within a social network is key to behavioral flexibility—Lessons from mice tested under seminaturalistic conditions
Source: Sci Adv. 2025 Jan 3;11(1):eadm7255. doi: 10.1126/sciadv.adm7255 (PMC11698118; doi:10.1126/sciadv.adm7255)
Supplement: Supplementary file 1 — Figs. S1 to S11 Table S1 [file sciadv.adm7255_sm.pdf]

Science Advances
AAAS

Supplementary Materials for

Information sharing within a social network is key to behavioral flexibility—Lessons from mice tested under seminaturalistic conditions

Maciej Winiarski *et al.*

Corresponding author: Ewelina Knapska, e.knapska@nencki.edu.pl; Alicja Puścian, a.puscian@nencki.edu.pl

Sci. Adv. **11**, eadm7255 (2025)
DOI: 10.1126/sciadv.adm7255

This PDF file includes:

Figs. S1 to S11
Table S1

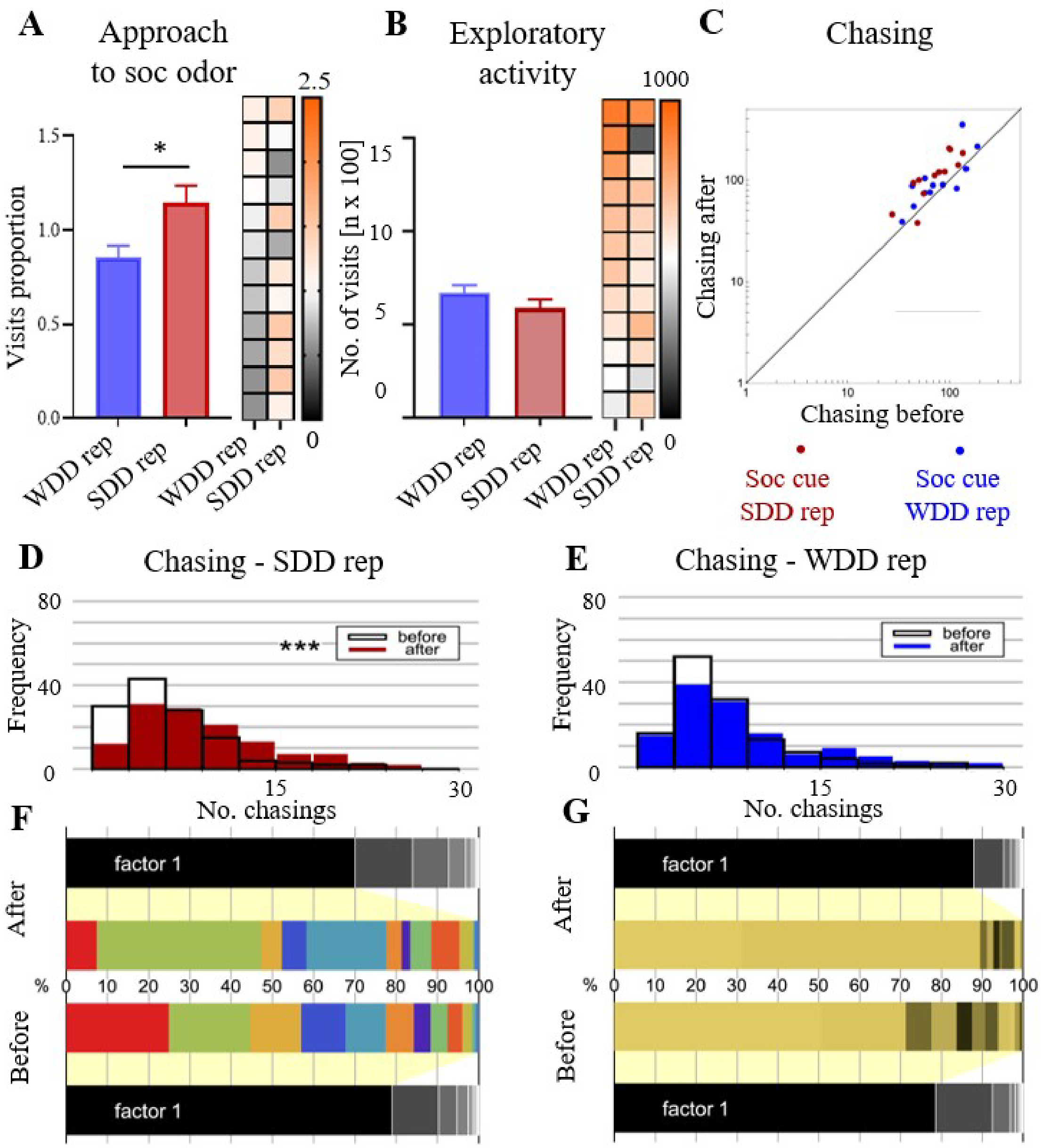

Fig S1. Replication of the experiment shown in Fig 1. Social olfactory cues indicating sucrose reward attract mice. (**A**) Mice prefer the compartment where the bedding soaked with the scent of the Sucrose-Drinking Demonstrator (SDD rep) was presented over the compartment where the bedding soaked with the scent of the Water-Drinking Demonstrator (WDD rep) was presented. (**B**) Social olfactory cues indicating reward availability do not change exploratory activity. Total number of visits to all compartments of the Eco-HAB is shown. Values for individual subjects are presented on the heatmap (right to the bar plots), squares in each row represent data for the same

mouse, and columns represent trials. Data in bar graphs are shown as mean +/- standard error. **(C-E)** The presence of the social olfactory cues in the SDD rep condition increases the number of chasings in the corridors of the Eco-HAB system. (**D**, **E**) Histograms show the distribution of chasings between all mice in the dark phases of the experiment (habituation period - before vs. period when the stimuli carrying social information were presented in the environment - after). (**F**, **G)** Spectral analysis of the social network. The size of the horizontal bars represents the extent to which each individual contributes to the composition of the main factor (factor 1) underlying shape of the network. Contributions of individual mice to factor 1 are represented with different colors. *$p<0.05$, ***$p<0.001$, **SDD rep - S**ucrose-**D**rinking **D**emonstrator replication, **WDD rep** – **W**ater-**D**rinking **D**emonstrator replication.

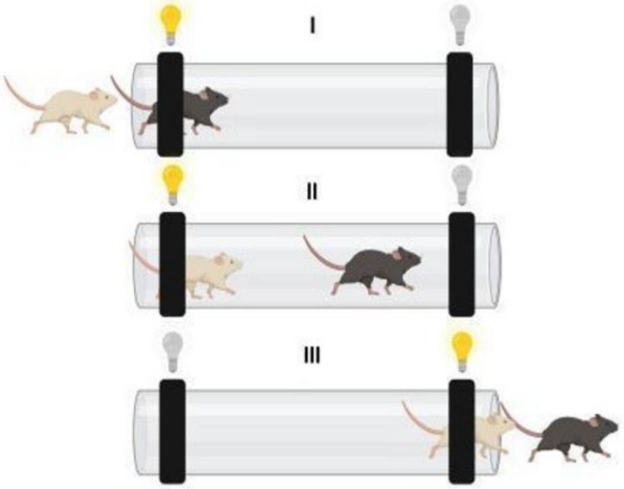

Fig S2. The exact order of antenna activations required for the behavior to meet the definition of chasing behavior. Chasing was defined as an event when one mouse entered a corridor (activation of the entrance antenna by mouse A, followed by another mouse (activation of the entrance antenna by mouse B) before the first left the tube (activation of the exit antenna by mouse A and subsequently by mouse B), and when both mice left the corridor in the same order and the same direction. Notably, the behavior was not categorized as chasing when the two animals backed up to the cage they originally came from or passed one another in the corridor.

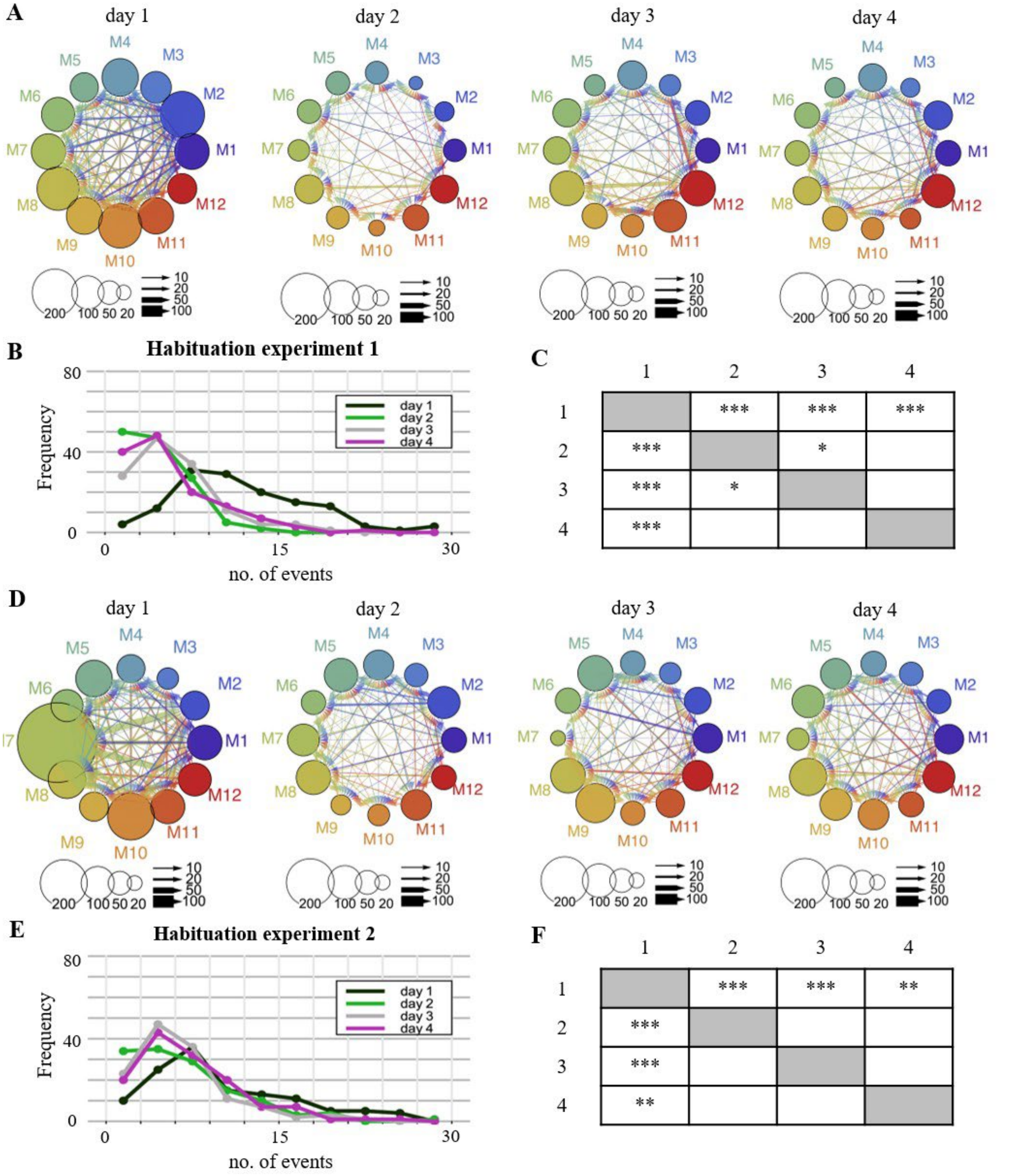

Fig S3.Observation of the social network in naïve animals (Habituation experiments 1 & 2) **shows that it is formed very early in the experiment and remains stable throughout**. (**A**) Social network of naïve mice housed in the Eco-HAB system without any additional stimulation

for 4 days. Patterns of chasing between individuals form a group's social network represented as a weighted, directed graph with nodes corresponding to individual mice and edges to interactions between them. Different colors represent the chasing of each mouse. The radius of the colored circle at a given node is proportional to the number of chasing performed by the corresponding mouse. The arrows are directed from a chaser to a chased individual; the thickness of an arrow is proportional to the number of chasing a given mouse performed after the other one. (**B**) Histogram shows the distribution of the number of following events in all pairs of mice within the cohort in the subsequent dark phases of the experiment (1-4). Subsequent days of the experiment are marked with different colors. (**C**) Table showing statistical comparisons between all days of the experiment (1-4) showing that social network stops changing after 3 days of habituation. (**D-F**) The same data is shown for the Habituation experiment 2. *p<0.05, **p<0.01, ***p<0.001.

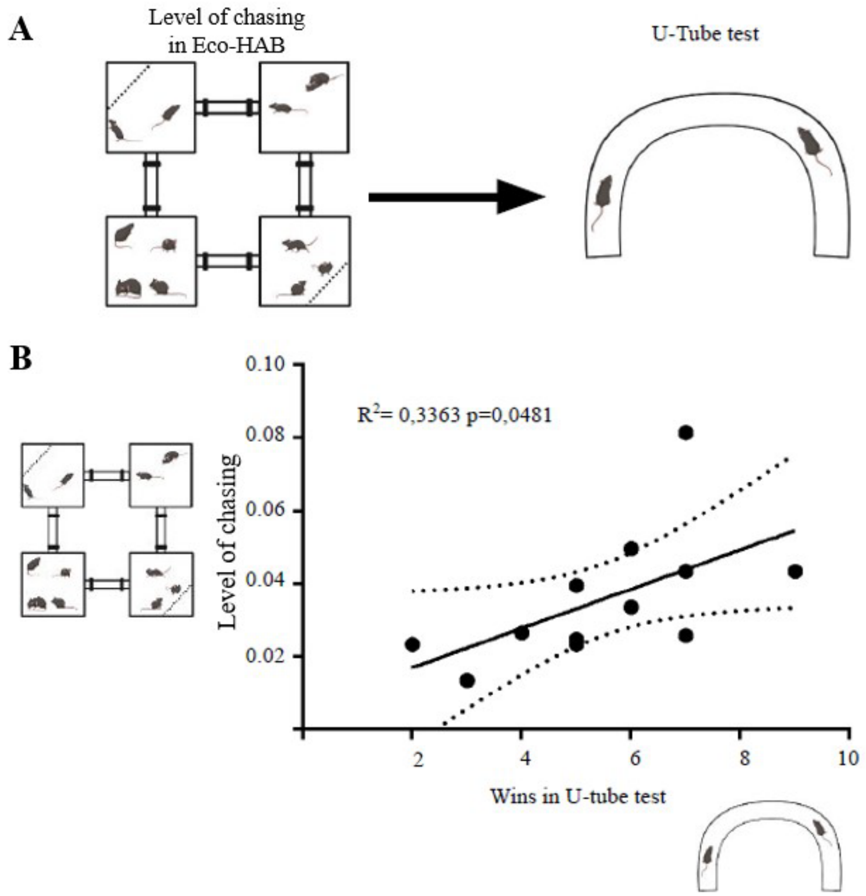

Fig S4. The number of chasings performed by individual mice corresponds with their social status. (A) Schematic of the experimental design. First, chasing behavior was measured in the Eco-HAB, and then the U-tube dominance test was performed. (**B)** Positive correlation between the position within the social network, defined by the number of chasing performed in the Eco-HAB, and dominance hierarchy as defined by the U-tube dominance test. The dominant mice were the ones chasing others the most.

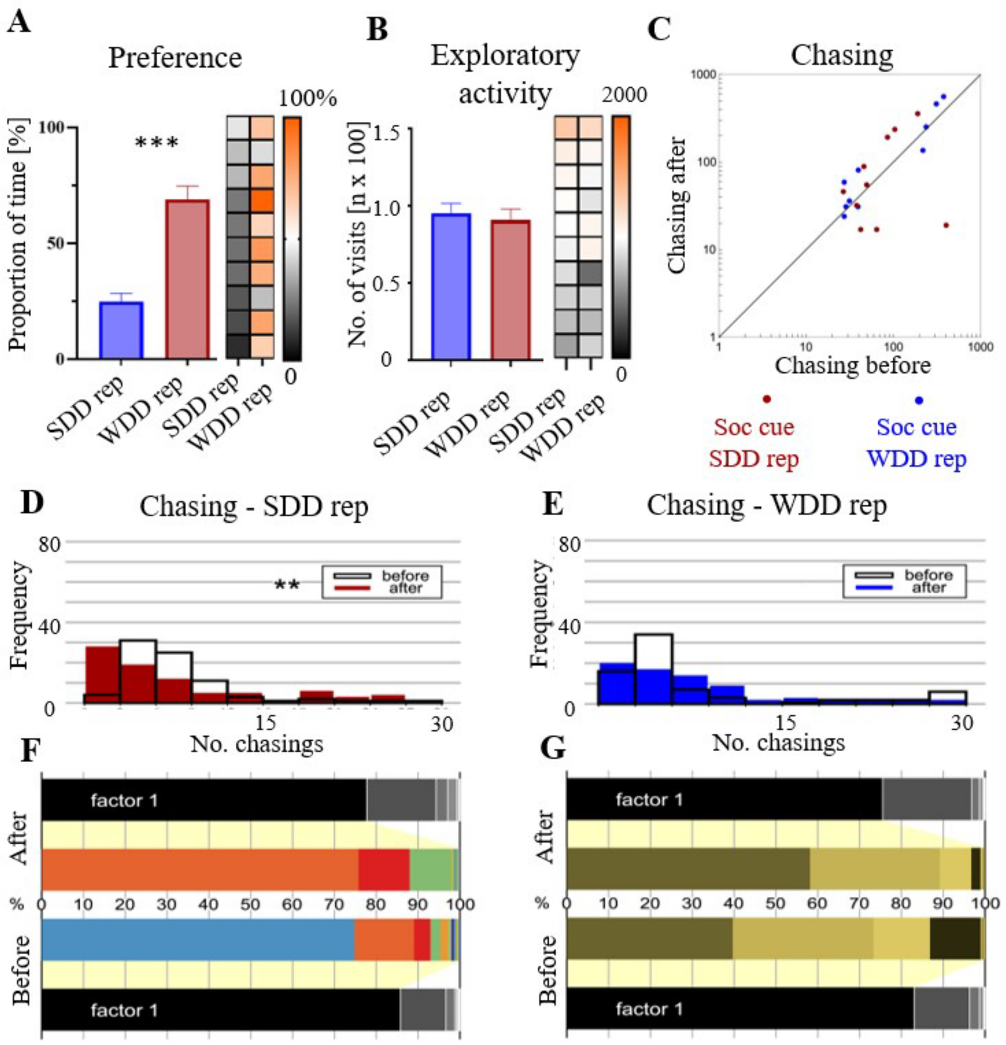

Fig S5. Replication of the experiment shown in Fig. 6. Social olfactory information helps to find the reward in a novel environment. (**A**) Newcomer mice prefer drinking from the bottle in the compartment where the scout mice were rewarded. (**B**) The exploratory activity is unchanged between the WDD-rep and SDD-rep conditions. Data in the bar graphs are shown as mean +/- standard error. Values for individual subjects in (**A-B**) are presented on the heat map (right to the bar plots) in accordance with the order of bars, sorted by the control. (**C**) The relation of chasing behavior during the habituation period (before) to chasing during the testing phase (after). (**D-E**) In the SDD-rep chasing intensifies upon the introduction to the new environment containing social cues indicating sucrose reward, which is not the case in the WDD-rep condition. (**F-G**) Spectral analysis of the social networks in WDD-rep and SDD-rep groups. **p<0.01, ***p<0.001.

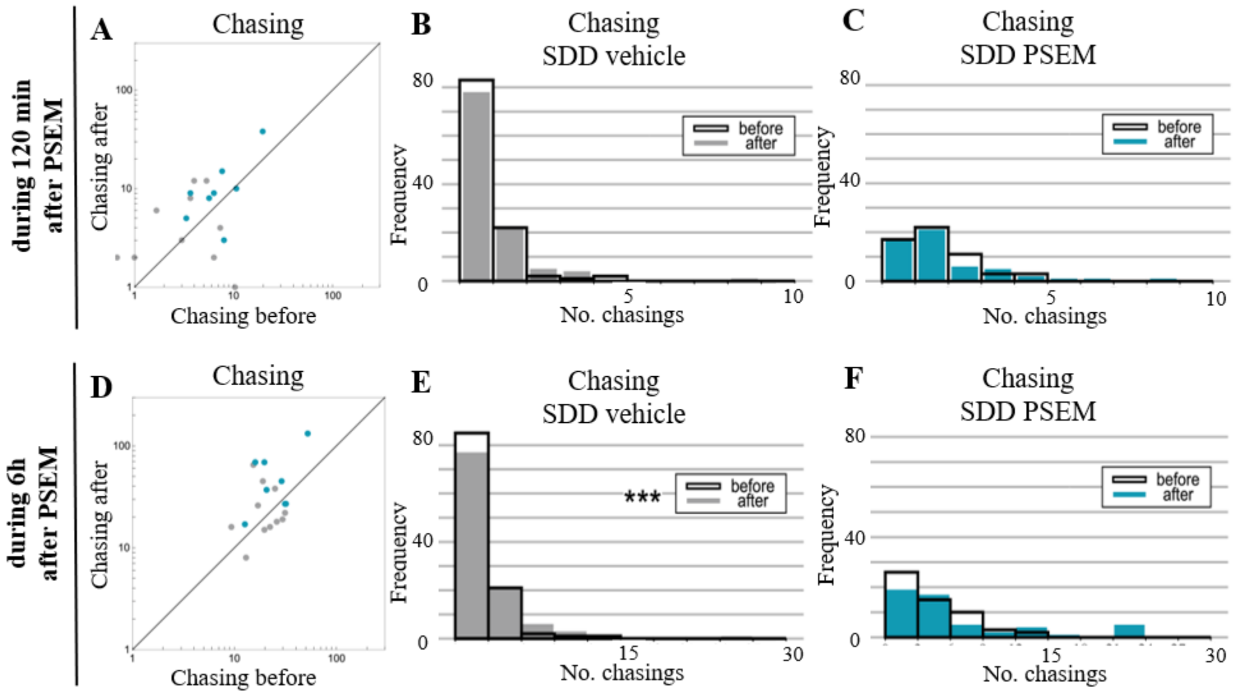

Fig S6. The direct impact of the chemogenetic intervention on chasing. (**A-C**) Chasing during the 2h period of neuronal manipulation (right after PSEM injection); such a short time is not sufficient for animals to show any changes in chasing patterns in response to the presentation of the social cue indicating sucrose reward in either of the tested groups (SDD vehicle, SDD PSEM). (**D-F**) The analysis of the broader, 6h period after the injections reveals that the intensifying of chasing starts appearing in the SDD vehicle condition, but not yet in the SSD PSEM condition. ***p<0.001.

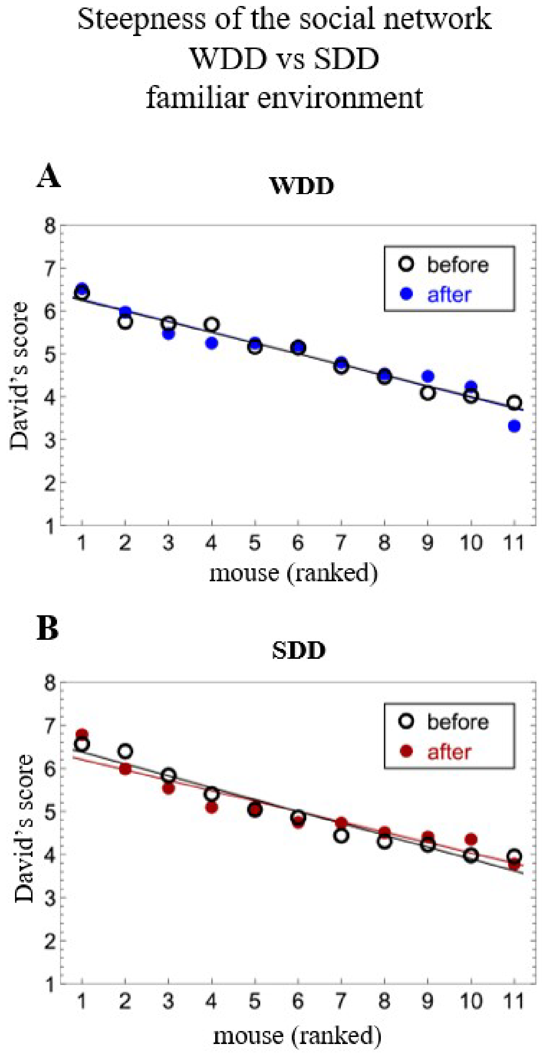

Fig S7. Steepness of a social network does not change after the presentation of social olfactory cues indicating sucrose reward. David's score of the mice before and after the presentation of the social cue in **(A)** the Water-Drinking Demonstrator (WDD) condition and **(B)** the Sucrose-Drinking Demonstrator (SDD) condition. Mice are ranked in accordance with their individual score, from top to bottom.

Steepness of the social network
WDD vs CDD
familiar environment

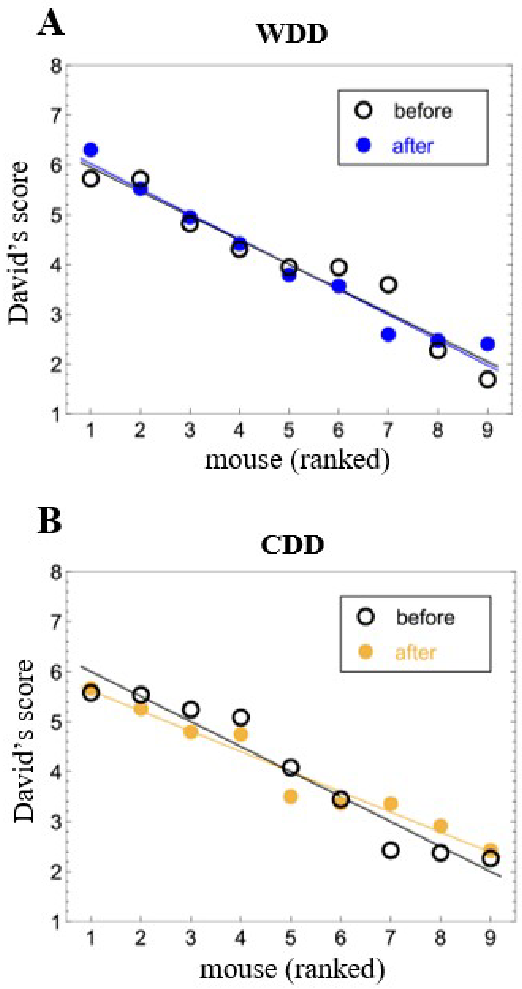

Fig S8. Steepness of a social network does not change after the presentation of social olfactory cues indicating cheese reward. David's score of the mice before and after the presentation of the social cue in **(A)** the Water-Drinking Demonstrator (WDD) condition and **(B)** the Cheese-Drinking Demonstrator (CDD) condition. Mice are ranked in accordance with their individual score, from top to bottom.

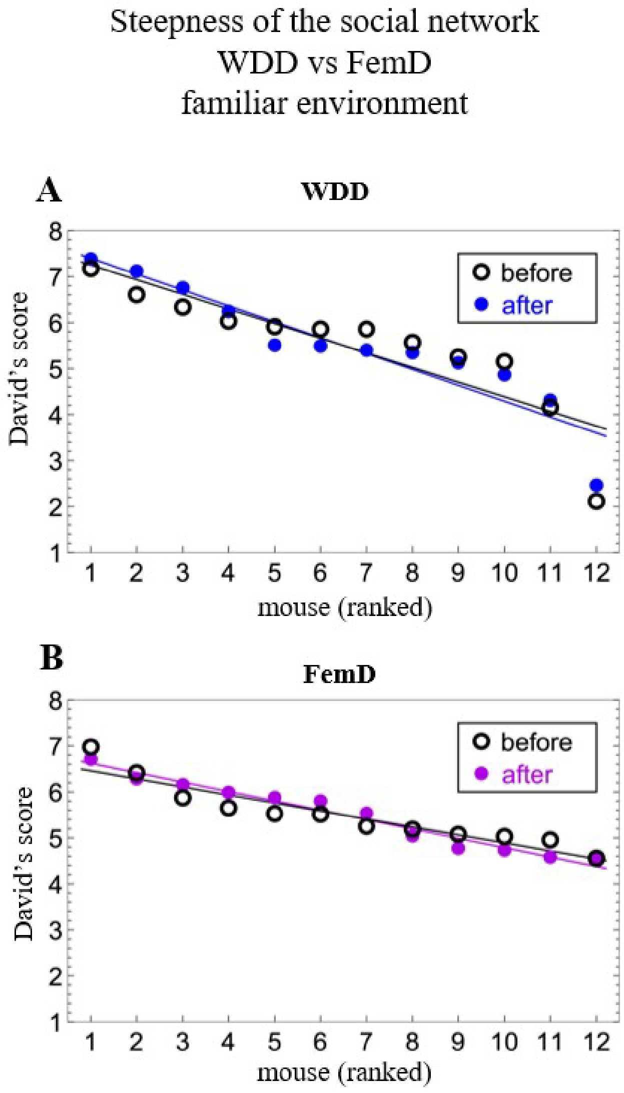

Fig S9. Steepness of a social network does not change after the presentation of social olfactory cues indicating exposure to female. David's score of the mice before and after the presentation of the social cue in **(A)** the Water-Drinking Demonstrator (WDD) condition and **(B)** the Female-Exposed Demonstrator (FemD) condition. Mice are ranked in accordance with their individual score, from top to bottom.

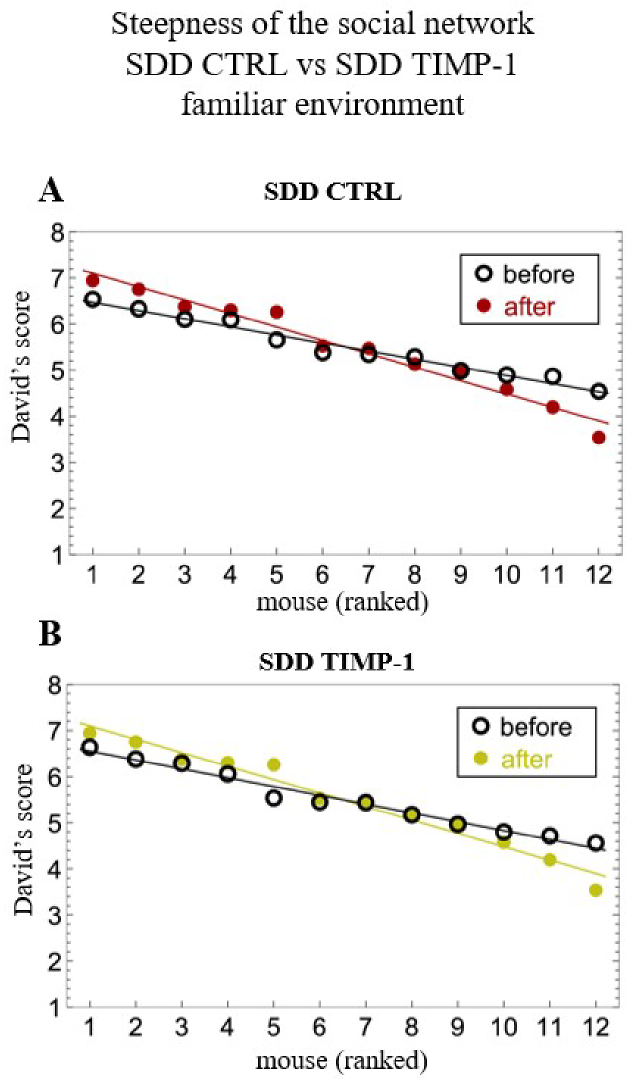

Fig S10. The Steepness of the social network in mice tested in the familiar environment and presented with the sucrose reward-indicating social cue does not change after the TIMP-1 injections into the PL. (A) David's score of the mice before and after the presentation of the social cue in the Sucrose-Drinking Demonstrator (SDD) condition before the brain manipulation. (**B**) The same score is shown for the cohort after the TIMP-1 injections. Mice are ranked in accordance with their individual score, from top to bottom.

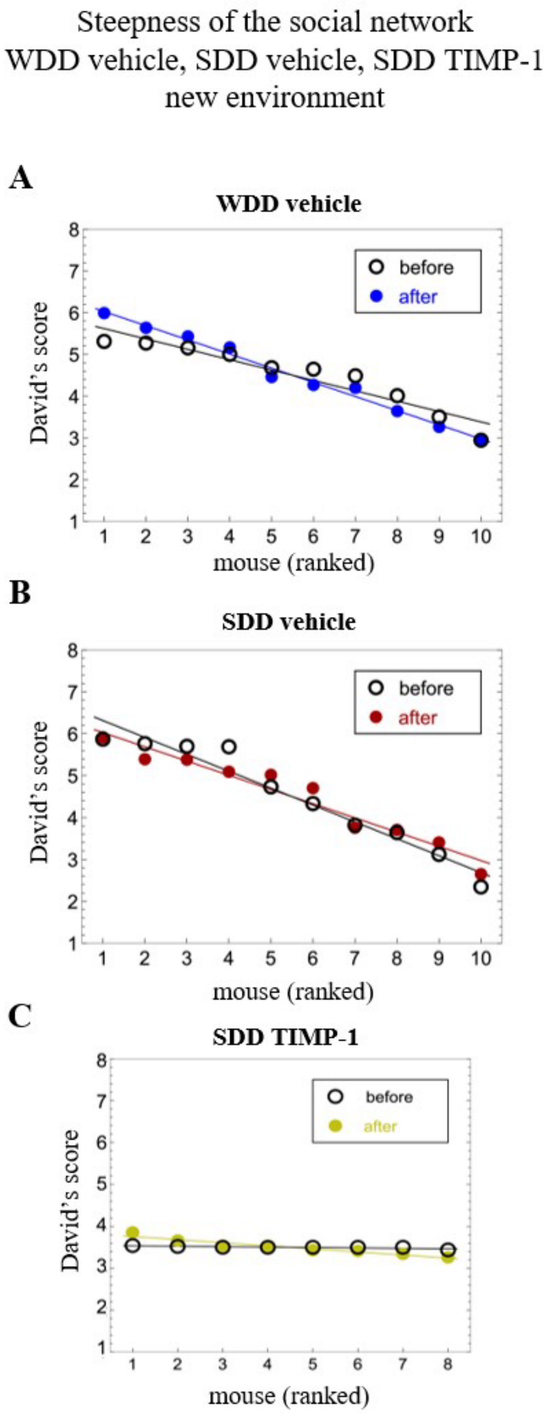

Fig S11. Steepness of the social network in mice tested in the novel environment and presented with the sucrose reward-indicating social cue does not change after the TIMP-1 injections into the PL. (A) David's score for the vehicle-injected mice before and after the presentation of the social cue from the Water-Drinking Demonstrator (WDD vehicle). (**B**) The same score is shown for the vehicle-injected mice presented with the social cue from the Sucrose-

Drinking Demonstrator (SDD vehicle) – before and after. (**C**) David's score before and after the stimuli presentation for the TIMP-1-injected mice exposed to the social cue from the Sucrose-Drinking Demonstrator (SDD TIMP-1). Mice are ranked in accordance with their individual score, from top to bottom.

Table S1. Data table

Figure	Comparison	Test	Statistic A	Value A	Statistic B	Value B	p-value
Fig.1B	Approach to social odor – WDD vs. Approach to social odor- SDD n=10	Paired t-test	Mean approach to odor	0.95 +/- 0.08	Mean approach to odor	1.31 +/- 0.12	0.006
Fig.1C	Exploratory activity - WDD vs. exploratory activity - SDD n =10	Paired t-test	Mean exploratory activity	635.2 +/- 72.7	Mean exploratory activity	808.1 +/- 109.9	0.113
Fig.2F	Histograms of Chasing SDD – testing phase vs. baseline n=45	Kolmogorov-Smirnov test	Frequency of chasing baseline	n/a	Frequency of chasing testing phase	n/a	<0.001
Fig.2G	Histograms of Chasing WDD – testing phase vs. baseline n=45	Kolmogorov-Smirnov test	Frequency of chasing baseline	n/a	Frequency of chasing testing phase	n/a	0.052
Fig.3B cheese	Approach to social odor – WDD vs. Approach to social odor- CDD n= 9	Wilcoxon matched-pairs signed rank test	Mean approach to odor	667.3 +/- 115.9	Mean approach to odor	676.3 +/- 86.93	>0.999
Fig.3C cheese	Exploratory activity - WDD vs. exploratory activity - CDD n = 9	Wilcoxon matched-pairs signed rank test	Mean exploratory activity	0.867 +/- 0.145	Mean exploratory activity	1.925 +/- 0.210	0.008
Fig.3E cheese	Histograms of Chasing WDD – testing phase vs. baseline n= 36	Kolmogorov-Smirnov test	Frequency of chasing baseline	n/a	Frequency of chasing testing phase	n/a	0.169
Fig.3F cheese	Histograms of Chasing CDD – testing phase vs. baseline n= 36	Kolmogorov-Smirnov test	Frequency of chasing baseline	n/a	Frequency of chasing testing phase	n/a	0.348
Fig.4B female	Approach to social odor – CTRL vs. Approach to social odor - FED n= 12	Wilcoxon matched-pairs signed rank test	Mean approach to odor	0.85 +/- 0.06	Mean approach to odor	1.23 +/- 0.13	0.012
Fig.4C female	Exploratory activity - CTRL vs. exploratory activity - FED n = 12	Wilcoxon matched-pairs signed rank test	Mean exploratory activity	669.2 +/- 41.7	Mean exploratory activity	588.3 +/- 46.7	0.310

Fig.4F female	Histograms of Chasing CTRL – testing phase vs. baseline n=55	Kolmogorov-Smirnov test	Frequency of chasing baseline	n/a	Frequency of chasing testing phase	n/a	0.087
Fig.4G female	Histograms of Chasing FED – testing phase vs. baseline n= 55	Kolmogorov-Smirnov test	Frequency of chasing baseline	n/a	Frequency of chasing testing phase	n/a	<0.001
Fig.4I female	Chasing WDD – testing phase n = 45 vs. Chasing SDD – testing phase n = 45	Kolmogorov-Smirnov test	Frequency of chasing testing phase	n/a	Frequency of chasing testing phase	n/a	0.026
Fig.4I female	Chasing WDD rep – testing phase n = 66 vs. Chasing SDD rep – testing phase n = 66	Kolmogorov-Smirnov test	Frequency of chasing testing phase	n/a	Frequency of chasing testing phase	n/a	<0.001
Fig.4I female	Chasing SDD – testing phase n = 45 vs. Chasing SDD rep – testing phase n = 66	Kolmogorov-Smirnov test	Frequency of chasing testing phase	n/a	Frequency of chasing testing phase	n/a	0.362
Fig.4I female	Chasing SDD – testing phase n = 45 vs. Chasing CDD – testing phase n = 36	Kolmogorov-Smirnov test	Frequency of chasing testing phase	n/a	Frequency of chasing testing phase	n/a	0.304
Fig.4I female	Chasing SDD – testing phase n = 45 vs. Chasing FED – testing phase n = 55	Kolmogorov-Smirnov test	Frequency of chasing testing phase	n/a	Frequency of chasing testing phase	n/a	< 0.001
Fig.4I female	Chasing CDD – testing phase n = 36 vs. Chasing SDD rep – testing phase n = 66	Kolmogorov-Smirnov test	Frequency of chasing testing phase	n/a	Frequency of chasing testing phase	n/a	0.128
Fig.4I female	Chasing CDD – testing phase n = 36 vs. Chasing FED – testing phase n = 55	Kolmogorov-Smirnov test	Frequency of chasing testing phase	n/a	Frequency of chasing testing phase	n/a	< 0.001
Fig.4I female	Chasing FED – testing phase n = 55 vs. Chasing SDD rep – testing phase n = 66	Kolmogorov-Smirnov test	Frequency of chasing testing phase	n/a	Frequency of chasing testing phase	n/a	< 0.001
Fig.5B	Approach to social odor – SDD CTRL vs. approach to social odor - SDD-	Paired t-test	Mean approach to odor	1.24 +/- 0.08	Mean approach to odor	1.1 +/- 0.11	0.357

	TIMP-1 n=11						
Fig.5C	Exploratory activity – SDD CTRL vs. exploratory activity SDD – TIMP-1 n = 11	Paired t-test	Mean exploratory activity	1086 +/- 95.0	Mean exploratory activity	1076 +/- 117.0	0.889
Fig.5E	Histograms of Chasing SDD-CTRL – testing phase vs. baseline n= 55	Kolmogorov-Smirnov test	Frequency of chasing baseline	n/a	Frequency of chasing testing phase	n/a	0.021
Fig.5F	Histograms of chasing SDD-TIMP1 – testing phase vs. baseline n= 55	Kolmogorov-Smirnov test	Frequency of chasing baseline	n/a	Frequency of chasing testing phase	n/a	0.922
Fig.6B	Preference for the bottle – WDD vehicle n = 9 vs. preference for the bottle – SDD vehicle n = 10	Unpaired t-test	Mean preference for the bottle (%)	29.0 +/- 7.2	Mean preference for the bottle (%)	82.1 +/- 5.5	<0.001
Fig.6B	Preference for the bottle – SDD vehicle n = 10 vs. preference for the bottle – SDD TIMP-1 n = 8	Unpaired t-test	Mean preference for the bottle (%)	82.1 +/- 5.5	Mean preference for the bottle (%)	61.6 +/- 4.4	0.013
Fig.6B	Preference for the bottle – WDD vehicle n = 9 vs. preference for the bottle – SDD TIMP-1 n = 8	Unpaired t-test	Mean preference for the bottle (%)	29.0 +/- 7.2	Mean preference for the bottle (%)	61.6 +/- 4.4	0.002
Fig.6C	Exploratory activity – WDD vehicle n = 9 vs. exploratory activity – SDD vehicle n = 10	Unpaired t-test	Mean exploratory activity	892.8 +/- 44.8	Mean exploratory activity	1035 +/- 81.2	0.157
Fig.6C	Exploratory activity – SDD vehicle n = 10 vs. exploratory activity – SDD TIMP-1 n = 8	Unpaired t-test	Mean exploratory activity	1035 +/- 81.2	Mean exploratory activity	545.4 +/- 65.2	<0.001
Fig.6C	Exploratory activity – WDD vehicle n = 9 vs. exploratory activity – SDD TIMP-1 n = 8	Unpaired t-test	Mean exploratory activity	892.8 +/- 44.75	Mean exploratory activity	545.4 +/- 65.15	<0.001
Fig.6E	Histograms of chasing WDD vehicle – testing phase vs. baseline	Kolmogorov-Smirnov test	Frequency of chasing baseline	n/a	Frequency of chasing testing phase	n/a	0.196

	n= 36						
Fig.6F	Histograms of chasing SDD vehicle – testing phase vs. baseline n= 45	Kolmogorov-Smirnov test	Frequency of chasing baseline	n/a	Frequency of chasing testing phase	n/a	0.045
Fig.6G	Histograms of chasing SDD TIMP-1 – testing phase vs. baseline n= 28	Kolmogorov-Smirnov test	Frequency of chasing baseline	n/a	Frequency of chasing testing phase	n/a	<0.001
Fig.7B	Preference for the bottle - SDD-vehicle n = 10 vs. Preference for the bottle – SDD-PSEM n = 7	Mann-Whitney U-test	Mean preference for the bottle (%)	54.7 +/- 4.5	Mean preference for the bottle (%)	31.3 +\- 5.7	0.007
Fig.7C	Exploratory activity SDD-vehicle n = 10 vs. exploratory activity SDD-PSEM n = 7	Mann-Whitney U-test	Mean exploratory activity	118.7 +/- 9.8	Mean exploratory activity	203.3 +/- 71.6	0.364
Fig.7E	Histograms of chasing SDD vehicle– testing phase vs. baseline 6-12h after PSEM n=55	Kolmogorov-Smirnov test	Frequency of chasing baseline	n/a	Frequency of chasing testing phase	n/a	0.028
Fig.7F	Histograms of chasing SDD PSEM – testing phase vs. baseline 6-12h after PSEM n=28	Kolmogorov-Smirnov test	Frequency of chasing baseline	n/a	Frequency of chasing testing phase	n/a	<0.001
Fig.S1A	Approach to social odor – WDD rep familiar environment vs. approach to social odor – SDD rep familiar environment n = 12	Paired t-test	Mean approach to odor	0.85 +/- 0.06	Mean approach to odor	1.15 +/- 0.09	0.050
Fig.S1B	Exploratory activity - WDD rep familiar environment vs. exploratory activity - SDD rep familiar environment n = 12	Wilcoxon matched-pairs signed rank test	Mean exploratory activity	669.2 +/- 41.7	Mean exploratory activity	590.2 +/- 43.8	0.092
Fig.S1D	Histograms of chasing SDD rep familiar environment –	Kolmogorov-Smirnov test	Frequency of chasing baseline	n/a	Frequency of chasing testing phase	n/a	<0.001

	testing phase vs. baseline n=66						
Fig.S1E	Histograms of chasing WDD rep familiar environment – testing phase vs. baseline n= 66	Kolmogorov-Smirnov test	Frequency of chasing baseline	n/a	Frequency of chasing testing phase	n/a	0.087
Fig.S4B	Correlation chasing in Eco-HAB vs. wins in U-tube test n=12	Pearson correlation	Chasing	Not applicable	Wins	Not applicable	0.048
Fig. S5A	Preference for the bottle – WDD rep new environment vs. preference for the bottle – SDD rep new environment n = 10	Paired t-test	Mean preference for the bottle (%)	24.9 +/- 3.5	Mean preference for the bottle (%)	68.9 +/- 5.8	<0.001
Fig. S5B	Exploratory activity – WDD rep new environment vs. exploratory activity – SDD rep new environment n = 10	Paired t-test	Mean exploratory activity	951.6 +/- 63.7	Mean exploratory activity	906.4 +/- 71.7	0.430
Fig. S5D	Histograms of chasing SDD rep new environment – testing phase vs. baseline n= 45	Kolmogorov-Smirnov test	Frequency of chasing baseline	n/a	Frequency of chasing testing phase	n/a	0.006
Fig. S5E	Histograms of chasing WDD rep new environment – testing phase vs. baseline n= 45	Kolmogorov-Smirnov test	Frequency of chasing baseline	n/a	Frequency of chasing testing phase	n/a	0.163
Fig. S6B	Histograms of chasing SDD vehicle– testing phase vs. baseline 0-2h after PSEM n=55	Kolmogorov-Smirnov test	Frequency of chasing baseline	n/a	Frequency of chasing testing phase	n/a	0.531
Fig. S6C	Histograms of chasing SDD PSEM – testing phase vs. baseline 0-2h after PSEM n=28	Kolmogorov-Smirnov test	Frequency of chasing baseline	n/a	Frequency of chasing testing phase	n/a	0.659
Fig. S6E	Histograms of chasing SDD vehicle– testing	Kolmogorov-Smirnov test	Frequency of chasing baseline	n/a	Frequency of chasing testing phase	n/a	<0.001

	phase vs. baseline 0-6h after PSEM n=55						
Fig. S6F	Histograms of chasing SDD PSEM – testing phase vs. baseline 0-6h after PSEM n=28	Kolmogorov-Smirnov test	Frequency of chasing baseline	n/a	Frequency of chasing testing phase	n/a	0.133
Fig. S7A	Steepness of the social network WDD familiar environment n = 11	as in de Vries H. et al., 2006 (87) see Methods for details	Steepness before	0.25 +/- 0.03	Steepness after	0.25 +/- 0.06	0.962
Fig. S7B	Steepness of the social network SDD familiar environment n = 11	as in de Vries H. et al., 2006 (87) see Methods for details	Steepness before	0.28 +/ -0.05	Steepness after	0.24 +/- 0.06	0.753
Fig. S8A	Steepness of the social network WDD familiar environment n = 12	as in de Vries H. et al., 2006 (87) see Methods for details	Steepness before	0.49 +/- 0.11	Steepness after	0.51 +/- 0.08	0.318
Fig. S8B	Steepness of the social network CDD familiar environment n = 8	as in de Vries H. et al., 2006 (87) see Methods for details	Steepness before	0.50 +/- 0.12	Steepness after	0.40 +/- 0.08	0.057
Fig. S9A	Steepness of the social network WDD familiar environment n = 12	as in de Vries H. et al., 2006 (87) see Methods for details	Steepness before	0.32 +/- 0.12	Steepness after	0.35 +/ 0.09	0.429
Fig. S9B	Steepness of the social network FED familiar environment n = 12	as in de Vries H. et al., 2006 (87) see Methods for details	Steepness before	0.17 +/- 0.05	Steepness after	0.20 + 0.03 – 0.02	0.977
Fig. S10A	Steepness of the social network SDD CTRL familiar environment n = 11	as in de Vries H. et al., 2006 (87) see	Steepness before	0.18 +/- 0.03	Steepness after	0.29 +/- 0.04	0.547

		Methods for details					
Fig. S10B	Steepness of the social network SDD TIMP-1 familiar environment n = 11	as in de Vries H. et al., 2006 (87) see Methods for details	Steepness before	0.15 +/- 0.02	Steepness after	0.21 +/- 0.07	0.663
Fig. S11A	Steepness of the social network WDD vehicle new environment n = 9	as in de Vries H. et al., 2006 (87) see Methods for details	Steepness before	0.25 +/- 0.07	Steepness after	0.34 +/- 0.03	0.052
Fig. S11B	Steepness of the social network SDD vehicle new environment n = 10	as in de Vries H. et al., 2006 (87) see Methods for details	Steepness before	0.40 +/- 0.08	Steepness after	0.34 +/- 0.07	0.346
Fig. S11C	Steepness of the social network SDD TIMP-1 new environment n = 8	as in de Vries H. et al., 2006 (87) see Methods for details	Steepness before	0.010 +/- 0.007	Steepness after	0.074 +/- 0.021	0.971
